# Identification and annotation of conserved promoters and macrophage-expressed genes in the pig genome

**DOI:** 10.1186/s12864-015-2111-2

**Published:** 2015-11-18

**Authors:** Christelle Robert, Ronan Kapetanovic, Dario Beraldi, Mick Watson, Alan L. Archibald, David A. Hume

**Affiliations:** The Roslin Institute and Royal (Dick) School of Veterinary Studies, University of Edinburgh, Easter Bush, EH25 9RG Edinburgh, UK; Institute for Molecular Bioscience, The University of Queensland, Brisbane, QLD 4072 Australia; Cancer Research UK, Cambridge Research Institute, Li Ka Shing Center, Robinson Way, Cambridge, CB2 0RE UK; Edinburgh Genomics, University of Edinburgh, Easter Bush, Edinburgh, EH25 9RG UK

**Keywords:** CAGE, FANTOM5, Promoters, Conservation, Pig, Macrophages, RNA-Seq

## Abstract

**Background:**

The FANTOM5 consortium used Cap Analysis of Gene Expression (CAGE) tag sequencing to produce a comprehensive atlas of promoters and enhancers within the human and mouse genomes. We reasoned that the mapping of these regulatory elements to the pig genome could provide useful annotation and evidence to support assignment of orthology.

**Results:**

For human transcription start sites (TSS) associated with annotated human-mouse orthologs, 17% mapped to the pig genome but not to the mouse, 10% mapped only to the mouse, and 55% mapped to both pig and mouse. Around 17% did not map to either species. The mapping percentages were lower where there was not clear orthology relationship, but in every case, mapping to pig was greater than to mouse, and the degree of homology was also greater. Combined mapping of mouse and human CAGE-defined promoters identified at least one putative conserved TSS for >16,000 protein-coding genes. About 54% of the predicted locations of regulatory elements in the pig genome were supported by CAGE and/or RNA-Seq analysis from pig macrophages.

**Conclusions:**

Comparative mapping of promoters and enhancers from humans and mice can provide useful preliminary annotation of other animal genomes. The data also confirm extensive gain and loss of regulatory elements between species, and the likelihood that pigs provide a better model than mice for human gene regulation and function.

**Electronic supplementary material:**

The online version of this article (doi:10.1186/s12864-015-2111-2) contains supplementary material, which is available to authorized users.

## Background

Research on pigs has important socio-economic impacts, including underpinning and accelerating improvements in the animal sector of agriculture [1], improving animal health and welfare and contributing to medical research by providing animal models [2, 3]. Understanding the relationship between genotype (sequence) and phenotype is critical to the use of pigs both in agriculture and in medical research. A high quality annotated reference genome sequence is an essential resource for contemporary research to understand the genetic control of complex traits.

The capability to sequence animal genomes at modest cost is now well-established but the assembly of whole genome shotgun sequence data into highly contiguous genome sequences remains non-trivial. Although a draft reference pig genome sequence has been generated [4], this resource has some limitations, including imperfect assembly and incomplete annotation. For example, the complexity of the pig transcriptome is significantly under-estimated. The current Ensembl annotation (Ensembl release 79) of the Sscrofa10.2 assembly recognises 30,585 different transcripts for the 24,754 protein coding and non-coding RNA genes yielding a ratio of ~1.2 transcripts per gene. The better-characterised human and mouse genomes yield ratios of annotated transcripts per gene of 4.4 and 3.0, respectively. Therefore the current annotation of the pig genome might not reflect the true complexity of the pig transcriptome, including alternative promoters, initiation and splicing. There are other significant gaps in the annotation of the pig genome that limit efforts to associate sequence with function, including knowledge of transcription start sites (TSS), promoters, enhancers and other functional elements.

The aim of the recently launched international Functional Annotation of Animal Genomes (FAANG) initiative [5] is to improve genome annotation for a range of animals, including the pig. The FAANG project will involve a major effort to generate species-specific information for functional annotation in a manner analogous to the Encyclopedia of DNA Elements (ENCODE) and mouse ENCODE projects [6, 7]. In advance of this effort, we asked whether functional annotation can be projected from well-characterised genomes of humans and mice. Deep sequencing of Cap Analysis of Gene Expression (CAGE) libraries allows the identification of transcription start sites , and hence promoters, and also a significant proportion of active enhancers which can be detected through their generation of bidirectional enhancer RNAs (eRNAs) [8, 9]. In the present paper, we combine cross-species mapping of defined promoters from human and mouse with RNA sequencing (RNA-Seq) and CAGE data derived from pig macrophages. It was already evident from low coverage shotgun sequencing of the pig that the 5’UTR regions were much more conserved between pig and human than between pig and mouse [10]. The pig genome sequence paper did not undertake an analysis of promoter-associated variation, because the transcription start sites were not defined. Within the Functional Annotation of Mammalian Genomes 5 (FANTOM5) consortium, we have noted that single nucleotide variants (SNVs) that are associated with disease susceptibility are strongly enriched within the −300 to +100 window surrounding TSS [8], perhaps reflecting the fact that these regions must be in open chromatin and are flanked by a positioned nucleosome [11]. The data also highlight those areas of the draft pig genome that require additional sequencing and assembly.

## Results

### Identification of conserved promoters in the pig

In a systematic comparison of CAGE-defined mouse promoters with the promoters of human and dog orthologous genes, the average level of conservation peaked around the TSS at around 85% in rat and 65% in human, and declined rapidly towards the genomic background at around −300 and +100 relative to the TSS [9]. Additionally Pol2 ChIP-Seq binding occupancy signal was observed mainly within a 500 bp window around the main TSS of all CAGE-derived human promoters [9]. Based on these observations, an extended genomic window of 501 bp was selected around the main human CAGE-defined TSS (see methods).

In a previous study, we compared the promoters of macrophage-expressed genes that were expressed differentially between human, mouse and pig [12]. In several cases, Pustell alignments (similarity matrices) revealed that conservation in proximal promoters was interrupted by substantial short repeat element insertions which could confound alignment scores. Accordingly, to seek orthologous promoters in pigs and mouse based upon the FANTOM5 human CAGE data, we first focused on known orthologous regions between human/pig and human/mouse and mapped the [−400,+100] from the human CAGE-defined promoter regions onto the predicted orthologous region. Those CAGE-defined promoters that were not mapped to known orthologous regions were then mapped to the whole RepeatMasked target genome (pig or mouse) using blastn to find the best match (pig or mouse genomes) (see methods and Additional file 1: Figure S1).

**Fig. 1:**
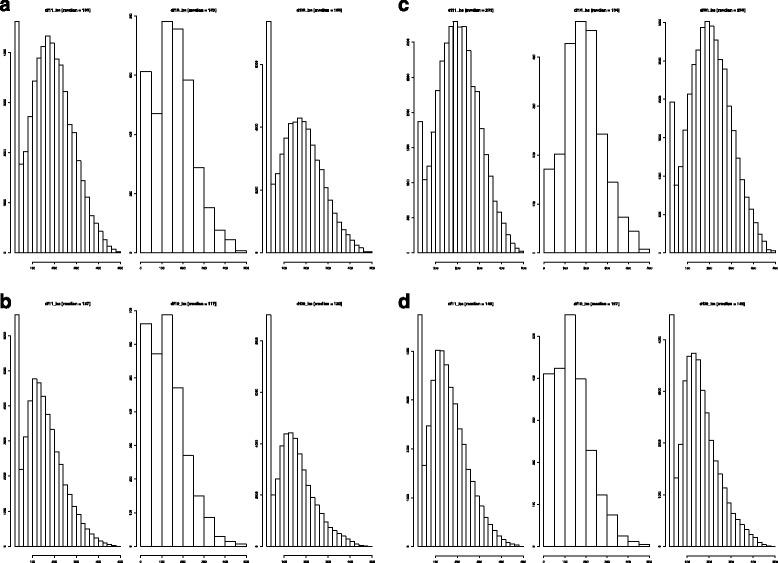
Distributions of bit-scores for the mapped FANTOM5 human promoters to the pig and mouse genomes**.** Distribution of bit-scores based on the mappings of FANTOM5 human promoters in the pig and mouse genomes. The distributions are based on different subset of FANTOM5 human promoters: (1a) set of promoters only mapped to the pig genome, (1b) set of promoters only mapped to the mouse genome, (1c) set of promoters mapped to pig and also present in the mappings to the mouse genome and (1d) set of promoters mapped to mouse and also present in the mappings to the pig genome. Class df11 (df10): represents the set of FANTOM5 human promoters for which there is an associated human gene with (without) a mouse ortholog respectively. Class df00: corresponds to the FANTOM5 human promoters lacking an associated gene ID

We separately analysed classes in which the human promoter was associated with an EntrezGene ID, with (referred to as class 11) or without (referred to as class 10) a putative ortholog, or not associated at all with a gene name (referred to as class 00). The number of FANTOM5 promoters and genes in each class are summarized in Table 1. As shown in Figs. 1a, b, c, d (bit-score distributions for pig-specific genes, mouse-specific genes and genes identified with human promoters mapping to both species [1c: pig, 1d: mouse]), the median of the bit-score distribution for the set of pig-specific genes is higher over all promoter classes than the mouse-specific genes. When comparing bit-scores between the mapping of human promoters to the pig and mouse genomes for the set of human promoters that mapped to both species, a similar trend emerges whereby all the bit-score medians are significantly higher in the mapping to the pig genome compared to the mouse across all classes (Fisher’s exact test P-value < 2.2e-16). The method used for the mapping of promoters across species is therefore consistent with the overall known higher conservation between human and pig versus human and mouse in various distinct genomic regions including intergenic regions where the vast majority of promoters are located [13].

**Table 1:** Mapping statistics for FANTOM5 human promoters

ID	M.O.	#Promoters	#Human	#Promoters	#Human	#Promoters	#NotReported	Total	Total
		Pig-specific	Genes(1)	Mouse-specific (2)	Genes(2)	Both	Promoters	#Promoters	#Genes
		(1)							
X	X	13033 [17%]	6215 [39%]	7945 [10%]	3483 [21%]	41606 [55%]	12503 [17%]	75087	15847
X	-	1539 [22%]	754 [24%]	700 [10%]	364 [11%]	2256 [32%]	2473 [35%]	6968	3059
-	-	19621 [19%]	-	11045 [10%]	-	37167 [36%]	34939 [34%]	102772	-
Total		34193 [19%]	6969 [37%]	19690 [11%]	3847 [20%]	81029 [44%]	49915 [27%]	184827	18906

Table shows the statistics regarding the mapping of the FANTOM5 human promoters to two target genomes. The number of human promoters mapped specifically and unequivocally to pig or mouse are indicated in columns #Promoters Pig-specific and #Promoters Mouse-specific respectively for distinct gene subsets: (i) human promoters which were assigned an associated EntrezGene ID (as defined in [9] *i.e.* within at most 500 bp of the 5’ end of the gene and located on the same strand) for which a known mouse ortholog exists (first row), (ii) those promoters with an associated gene but no known mouse ortholog (2^nd^ row) and (iii) promoters for which there is no gene association within the 500 bp window (3^rd^ row). (1) and (2) refer to species pig and mouse respectively. M.O. stands for mouse ortholog. A cross indicates the presence of the given feature *i.e.* presence of an associated EntrezGene ID (column ID) or a known mouse ortholog (column M.O.). Numbers of human genes associated to a given set of mapped promoters are indicated in columns denoted #HumanGenes when applicable. The columns entitled ‘#Promoters both’ and ‘#NotReported promoters’ show the number of promoters mapped to both species and the number of promoters not reported (including unmapped and multimapped promoters) respectively. All percentages are indicated in square brackets. The final two columns show the total number of promoters and genes for each category considered (*i.e.* each row)

Table 1 shows the results of the mapping of the 184,827 high confidence human promoters from FANTOM5 -*i.e.* robust set defined in [9]- to the pig (Sscrofa10.2) and mouse (mm9) genomes. For those genes in which the human TSS was linked to an Entrez Gene ID with a putative mouse ortholog, 17% mapped only to the pig genome but not to the mouse, 10% mapped only to the mouse, and 55% mapped to both pig and mouse. Around 17% did not map to either species. For the smaller category of genes where there was no apparent ortholog of the human Entrez Gene ID in the mouse, the relative proportions of promoter conservation were proportionately higher in pig (with 22% mapped only to pig) and similar in mouse (with 10% mapped only to mouse), and 32% to both species. The presence of a conserved promoter region could provide additional evidence of orthology where it is not evident or equivocal based upon the protein coding sequence, or where there is inadequate assembly or annotations. The recent publication of the draft pig genome [4] revealed only around 9,000 1:1 orthologs across multiple mammalian species. Additional file 2: Tables S2A and S2B provides lists of the human Entrez Gene IDs, the locations of the mapped promoters in the pig (Additional file 2: Table S2A) and mouse (Additional file 2: Table S2B) genomes, and any annotation/name of the nearest downstream gene.

The set of genes associated with human FANTOM5 promoters mapping exclusively to the pig is significantly enriched for the Gene Ontology (GO) term “defense response to other organism” (Benjamini and Hochberg-corrected P-value of 3.88E-3). These include many of the genes that are induced by lipopolysaccharide in human and pig macrophages, but not in mice, as described previously [12].

The third category of human promoters is the one where there is no associated FANTOM5 human Entrez Gene ID. Many of these are long and short non-coding RNAs, transcribed pseudogenes and retrotransposons, all of which have previously been analysed in detail in FANTOM5 and in earlier studies from the FANTOM consortium. Indirectly, the relative inability to map these promoters lacking an associated gene, which we would not expect to be highly-conserved, can be considered a control for the much higher proportion of mapping of the FANTOM5 promoters that do have an associated annotation. Still, the unequivocal mapping to the pig genome only (19%) was greater than to the mouse genome (10%), with 36% mapping to both species. Another subclass of the promoters that is not associated with an Entrez Gene ID may represent distal enhancers, which can be identified based upon bidirectional promoter activity [8]. We separately mapped the 501 bp surrounding these annotated putative human enhancers (robust set) to the pig genome. Of these putative enhancer sequences 39% mapped to a single locus each (single mappers) in the pig genome and 21% were both single mappers and unequivocally mapped only to the pig genome (*i.e.* with no match to the mouse genome). In contrast, and consistent with the subsequent analysis by the mouse ENCODE consortium [14], only 21% of human enhancers identified by the FANTOM5 consortium were single mappers on the mouse genome and only 6% were both single mappers and unique to the mouse genome (absent in the pig mappings).

Many human protein-coding genes have more than one promoter defined by separate CAGE-identified transcription start site clusters, as evident from the identification of >80,000 TSS for ca. 20,000 loci [9]. The FANTOM5 dataset identified at least one TSS associated with 94% of protein-coding genes. If we considered the non-redundant list of 19,831 Ensembl IDs (corresponding to 18,929 Entrez Gene IDs) for human, 80% (15,797) had at least one TSS that could be mapped to the pig genome. Hence, the human CAGE data mapping supports predicted TSS for 73% (15,797) of the predicted 21,630 protein-coding genes currently annotated in the pig genome.

### Reasons for non-uniquely mapped -including unmapped- human promoters

**Fig. 2:**
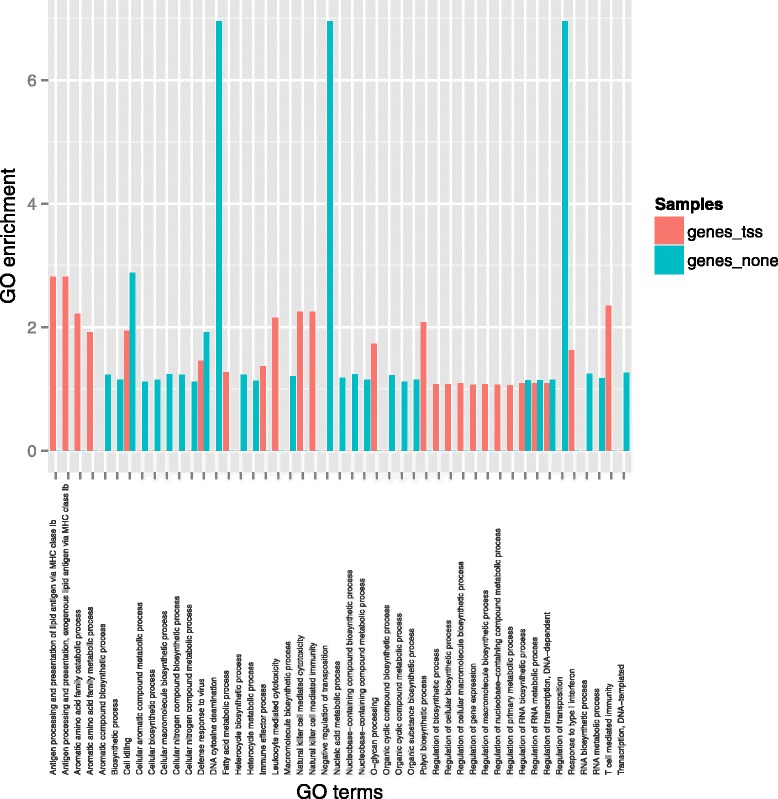
GO terms enrichment histograms for two sets of human genes. Histogram shows GO terms enrichment within two classes of human genes: (1) class with the set of Entrez Gene IDs of individual TSS that did not map to the pig genome (class referred to as genes_tss) and (2) separately of those that did not have at least one TSS mapped (class referred to as genes_none). GO enrichment analysis was performed against a background set consisting of all the human genes with a known associated FANTOM5 human TSS using GOrilla [54]

We next examined the reasons why a subset of the CAGE-defined human promoters did not map to the pig genome, and whether it might be possible to capture additional information from the FANTOM5 data. We first examined and dissected the non-uniquely mapped human TSS into those that that did not map at all (unmapped), and those that mapped equally to more than one location (multimapped). The latter category is likely to include multigene families, species-specific copy number variants and errors in the pig genome assembly. Table 1 dissects the TSS that did not map uniquely to the pig genome (*i.e.* set including unmapped and multimapped TSS) into categories. Only 17% (8,647/49,915) of TSS that did not uniquely map to the pig genome proved to be multimappers. Since multi-mapping CAGE tags were not rescued in FANTOM5 (in contrast to earlier studies which employed a rescue strategy [15]), this is not entirely surprising. To determine whether there might be a functional class of promoters that is rapidly-evolving and therefore not sufficiently conserved to permit mapping from human to pig, we performed GO analysis on the set of Entrez Gene IDs of individual TSS that did not map to the pig genome –*i.e.* the set of genes with at least one TSS unmapped- (referred to as genes_tss), and separately of those that did not have at least one TSS mapped –*i.e.* genes with all associated TSS unmapped (referred to as genes_none). Figure 2 shows a histogram of the GO terms of these two classes. The two classes of genes with unmapped promoters show weak GO term enrichments with slightly lower false discovery rate (FDR) adjusted q-value observed for the gene class for which none of the associated TSS mapped to the pig genome. The lists of GO terms for both gene classes are summarized along with the enrichment statistics in Additional file 3: Table S1. The gene class for which none of the associated promoters were mapped to the pig genome shows a weak enrichment (not statistically significant *i.e.* FDR adjusted q-value >1E-3) for genes involved in regulation of transposition. Transposition is known to play a major role in gene regulation and may lead to rapidly evolving genes and regulatory regions [16]. Amongst those regions, DNaseI-hypersensitive (HS) sites derived from transposable elements (TE) are known to be highly evolutionary divergent as opposed to non-TE derived HS sites in the human genome [17]. A lack of promoter conservation between human and pig species would be consistent with those highly divergent regulatory regions.

A second reason why a subset of human promoters did not uniquely map to the pig genome is that the current assembly contains duplications that may, or may not, reflect genuine copy number variations. We examined specifically the subset of multimappers that mapped at two locations only. There were 1,246 such examples for human to pig, and only 374 for human to mouse. Some of these may be genuine copy number variants (CNV) as described by others [18], although the majority of those were apparently non-genic [19]. Amongst these CNVs is the *c-KIT* locus, which is associated with coat color variation in some pigs [19], but the majority are olfactory receptors and duplicated more than 2x. One example discussed further below is the *CSF1R/PDGFRB* locus, part of which is duplicated in the current pig genome assembly. This region is an ancestral duplication related to *c-KIT/PDGFRA*, but was not amongst the duplicated genes detected in the previous study [19]. The apparent duplication is probably not genuine. The duplicated sequences are identical, and there is only one copy of the region in all other avian and mammalian genomes sequenced. The number of FANTOM5 mouse promoters that map only to two locations in the pig genome is lower (533), where a higher proportion (1886) map to two locations in the human genome. This pattern might be attributable to extensive copy number variation in the human genome that has not yet been subject to purifying selection [20–22] as well as the consequence of the incompleteness of the current pig genome assembly.

In the larger class of genes for which at least one promoter was not mapped to the pig genome, a weak enrichment (not statistically significant; FDR adjusted q-value of 2.48E-2) for genes involved in immune effector processes was observed. This observation would be in agreement with the rapid adaptation observed for promoters of genes involved in immune response – genes which are statistically over-represented among genes with TE-derived HS sites.

Finally, Table 1 revealed that at least some human TSS map to the mouse genome but not to the pig. As demonstrated below, this could be due in part to the fact that the pig genome assembly is not as mature as the mouse and human, and still contains many unplaced scaffolds. Table 2 shows the reciprocal mapping of the set of TSS derived from the FANTOM5 mouse CAGE data to pig and human genomes. The mouse data is derived from developmental states that were not readily assayed in humans, so the mouse and human are non-redundant in terms of state as well as genomic sequences. Of the 116,277 robust mouse CAGE-defined promoters, 48.8% are associated with an Entrez Gene IDs (for which there is at least one known Ensembl ID). In that subset of promoters, 8.3% mapped to the pig genome, but not to the human. This subset of mouse promoters -that mapped to the pig genome- was associated to 2,322 genes, we can infer therefore that the mouse CAGE data identify at least one candidate TSS annotation for 2,322 additional pig genes that have presumably diverged in the human genome. Additional file 4: Tables S3A and S3B provide the lists of the mouse Entrez Gene IDs, the locations of the mapped promoters in the pig (Additional file 4: Table S3A) and human (Additional file 4: Table S3B) genomes, and any Ensembl annotation of the nearest downstream gene when applicable.

**Table 2:** Mapping statistics for FANTOM5 mouse promoters

ID	M.O.	#Promoters	#Mouse	#Promoters	#Mouse	#Promoters	#NotReported Promoters	Total	Total
		Pig-specific	Genes(1)	Human-specific (2)	Genes(2)	Both		#Promoters	#Genes
		(1)							
X	X	4182 [8%]	2190 [13%]	7544 [14%]	3503 [22%]	22742 [44%]	16891 [33%]	51359	15777
X	-	526 [9%]	132 [5%]	655 [12%]	107 [4%]	1535 [29%]	2626 [49%]	5342	2629
-	-	5104 [8%]	-	6630 [11%]	-	20057 [34%]	27785 [47%]	59576	-
Total		9812 [8%]	2322 [13%]	14829 [13%]	3610 [20%]	44334 [38%]	47302 [41%]	116277	18406

Table shows the statistics regarding the mapping of the FANTOM5 mouse promoters to two target genomes. The number of mouse promoters mapped specifically and unequivocally to pig or human are indicated in columns #Promoters Pig-specific and #Promoters Human-specific respectively for distinct gene subsets: (i) mouse promoters which were assigned an associated EntrezGene ID (as defined in [9] *i.e.* within at most 500 bp of the 5’ end of the gene and located on the same strand) for which a known human ortholog exists (first row), (ii) those promoters with an associated gene but no known human ortholog (2^nd^ row) and (iii) promoters for which there is no gene association within the 500 bp window (3^rd^ row). (1) and (2) refer to species pig and human respectively. M.O. stands for murine gene with human ortholog. A cross indicates the presence of the given feature *i.e.* presence of an associated EntrezGene ID (column ID) or a known human ortholog (column M.O.). Numbers of mouse genes associated to a given set of mapped promoters are indicated in columns denoted #MouseGenes when applicable. The columns entitled ‘#Promoters both’ and ‘#NotReported promoters’ show the number of promoters mapped to both species and the number of promoters not reported (including unmapped and multimapped promoters) respectively. All percentages are indicated in square brackets. The final two columns show the total number of promoters and genes for each category considered (*i.e.* each row)

In total, the non-redundant list of Ensembl IDs from human (15,797) and mouse (9,839) that can be mapped to pig covers 16,828 predicted protein-coding genes (see methods).

If the FANTOM5 promoter mapping to the pig is accurate and informative, we would anticipate that the promoters of known protein-coding genes would map to the location of their orthologs. We therefore determined the FANTOM5 human promoters -with an associated Entrez gene ID- that mapped within a 20 Kb upstream of a defined ortholog (where such has been identified). Despite the proportionally higher unique mapping of human promoters to the pig genome noted above, the proportion of apparently appropriate locations was 81% (37,442/46,329) for human to pig, and 88% (44,106/49,991) for human to mouse mappings. The higher proportion of human to mouse mappings versus the human to pig mappings again reflects the variable level of completeness in genome assemblies – with the pig genome assembly being at an earlier stage of completion compared to the human and mouse genomes [23].

### Generation of pig CAGE and RNA-Seq libraries from macrophages

Many of the libraries sampled in the human and the FANTOM5 projects came from macrophages, and macrophages are amongst the most complex sources of mRNA [24]. We wished to validate the mapping of human TSS to the pig genome, and also to create a dataset in which it would be possible to compare precise TSS locations on specific genes using the macrophage as an example. We therefore prepared CAGE libraries from pig alveolar macrophages. In contrast to the human CAGE data derived from Helicos single molecule sequencing, the reads were sequenced using Illumina GA technology. Adapter, barcode and restriction enzyme sequences were trimmed and reads with a minimum of 20 bases were kept for further analysis (99% of sequences kept *i.e.* 3,856,071/3,899,341). We mapped reads both with and without quality trimming (using Q20 as a cut-off). The untrimmed data had a far higher percentage of uniquely mapped reads (49% vs. 20%). We believe the quality scores have been under-estimated by the Illumina base-calling software, due to the CAGE library being a low-diversity library [25, 26]. In order to avoid excluding false negatives (*i.e.* reads with under-estimated base qualities), no base quality filtering was applied prior to aligning the reads to the reference genome. The mapping statistics are summarized in Table 3: 61% of the reads were mapped (49% uniquely mapped and 12% as multimapped reads). Additionally the remaining unmapped reads (39%) were further trimmed from their 5’-end and 3’-end by one base and realigned as described in the method section. This approach resulted in the “rescue” of 20% of the originally unmapped reads with 31% remaining unmapped. Consequently, the percentage of reads mapped was increased to 69% with 56% as uniquely mapped reads and 13% as multimappers. These results are similar to equivalent mapping statistics for mouse FANTOM3 CAGE data [27].

**Table 3:** Mapping statistics for pig CAGE dataset

Library size	Library size	Total mapped	Uniquely mapped	Multimapped	Unmapped
(before trimming)	(after trimming)	to Sscrofa			
3899341	3856071	2655655	2145023	510632	1200416

Table showing: library sizes before and after trimming (see methods), total number of mapped pig CAGE tags to the pig genome (Scrofa10.2) and the number of uniquely mapped, multimapped and unmapped tags

Finally, we asked whether the complete set of macrophage-derived pig CAGE tags mapped into regions of the pig genome defined as likely promoters based upon the mapping of the FANTOM5 human promoters. The genomic locations where pig CAGE tags mapped to the pig genome were extended from both extremities –*i.e.* from both sides (5’/3’)- so that the length of the extended mapped region would correspond to that of the human FANTOM5 CAGE-derived promoters (*i.e.* 501 bases). The mappings of the human FANTOM5 CAGE-derived promoters were also extended following the same procedure. The set of extended uniquely mapped pig CAGE tags (2,145,023) was converted into CAGE-tag starting site clusters (CTSS) overlapping by at least one base (100,779 CTSSs). All CTSSs supported by only a single sequence read tag (44,157) were removed from further analysis. The remaining CTSSs with at least 2 tags (56,622) map in the upstream regions (2 Kb or within coding region) of 10,510 distinct genes representing 42% of the total set of known annotated porcine genes (10,510/25,322). Only 19% (10,695/56,622) to 28% (15,707/56,622) of these macrophage-associated clusters were proximal to a human FANTOM5-derived promoter at most 100 bases and 2 Kb away respectively. Conversely, the percentage of human FANTOM5 CAGE-derived promoters uniquely mapped to the pig genome that were associated with a mapped pig CAGE CTSS cluster ranges from 29% (d<=100 bases) to 42% (d<=2 Kb). Given that the pig CAGE data derived from a single cell type, albeit one that has a very diverse transcriptome, this represents a very high level of validation of predicted promoter locations based upon cross-mapping from the FANTOM5 data. We reasoned that the promoters expressed in human macrophages, mapped to the pig genome, would be even more likely to be supported by the pig macrophage CAGE data. This prediction is confirmed in Additional file 5: Figures S2A and S2B, where the analysis is restricted to the set of FANTOM5 promoters derived from the 55 human monocyte or macrophage libraries (see FANTOM5 library descriptions in Additional file 6: Table S4). We also looked at the sets of pig CAGE CTSS clusters with or without a nearby mapped human promoter, the set of CTSSs with an associated nearby human promoter contains a higher number of supporting CAGE tags – thus are more highly expressed than the set of CTSSs without a nearby promoter (see Additional file 7: Figure S3).

The vast majority (96%) of the pig CAGE tags that were unmapped to the pig genome (1,229,687) did not map to the human genome. None of the small proportion of pig CAGE tags that did align to specific genomic locations was found to be coincident with any human CAGE-defined TSS. These uniquely mapped CAGE reads (48,309) might correspond to new candidate TSS in human – not identified in the FANTOM5 project either due to their lack of expression in the cells/tissues/time-points studied or their failing the criteria to be added to the robust set of promoters [9].

Amongst the set of pig CAGE CTSS clusters that were multimapped to the pig genome- 7% (11,131) had at least one occurrence mapped in pig retro-transposons as annotated with either long/short interspersed nuclear element (LINE/SINE) or long terminal repeat (LTR) as the class of repeat (annotations extracted using the UCSC Table Browser for *Sus scrofa* 10.2 genome with selected group: variation and repeats, track: repeatmasker and table: rmsk) and 11% (16,291) of the multimapped CTSSs had at least one occurrence found overlapping any repeat from any class. Despite its small library size, the pig CAGE dataset used in this study provides some evidence for pig retro-transposon transcription. The remaining proportion of multimapped reads not rescued might be due to genuine gene duplication events or mis-assembled segmental duplications [23].

**Fig. 3:**
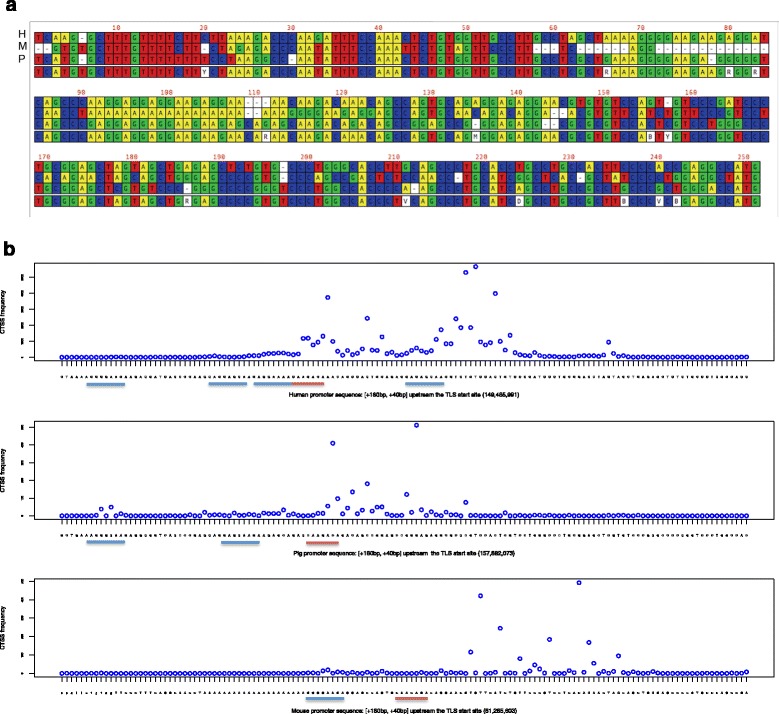
CTSS distribution at the aligned *CSF1R* locus across three species. Figure 3a shows the alignment of the three species at the *CSF1R* promoter region. H, M and P refer to human, mouse and pig species respectively. Figure 3b shows histograms of the CAGE tag locations in each of mouse, human and pig, aligned based upon the location of start codon (translation start site - TLS) downstream – region shown spans 180 bp upstream of the TLS and 40 bp downstream from it. Horizontal lines underline the binding sites of transcription factors PU.1 (blue lines) and EWS/FUS (red lines) [30]

Initiation of transcription for genes in which the promoter contains a TATA-box motif takes place at a precise location 30/31 bp downstream of the AT-rich sequence [28]. They are enriched amongst highly-inducible genes, and gain and loss of the TATA box between species can be associated with altered inducibility [29]. Most mammalian genes do not have a TATA box and instead initiate transcription in a broad window [28]. Previous comparative analysis of the promoters of mouse and human orthologous genes revealed that variation in the initiator, Pu/Py sequence can influence the precise TSS [28]. Most broad promoters are GC-rich, but macrophage-specific genes, exemplified by *CSF1R*, employ a novel purine-rich proximal promoter, regulated by the macrophage-specific transcription factor, PU.1 [30, 31]. Although there are some short conserved elements, the human *CSF1R* promoter used in macrophages does not map to the mouse. The species differ in that humans use an alternative promoter, 25 kb upstream of the macrophage promoter in the 3’UTR of *Pdgfrb*, to drive expression in placental trophoblasts, whereas the trophoblast promoter in mouse lies immediately upstream of the macrophage TSS [32]. To validate the pig macrophage CAGE data, we examined the precise location of the macrophage-specific *CSF1R* promoter. The human *CSF1R* promoters mapped to two adjacent locations in the pig genome, with identical sequences, and accordingly, there was no coverage of the pig macrophage *CSF1R* promoter by single-mapped tags. We therefore examined multi-mapped tags mapped to the duplicated promoter region. Figure 3A shows alignment of the three species, and Fig. 3B shows histograms of the CAGE tag locations in each of mouse, human and pig, aligned based upon the location of start codon which is located around 50 bp downstream of the TSS cluster in the first exon. The greater depth of FANTOM5 CAGE data, compared to earlier analysis [30], revealed that human *CSF1R* transcription initiates from two peaks, associated with a duplication of the proximal promoter elements; the binding sites for PU.1 and the EWS/FUS [30]. The mouse promoter does not contain this duplication, and initiates in a single peak that is weakly aligned to the human, but contains the same EWS motif. The pig promoter also does not contain the duplicated proximal elements, but the TSS is coincident with the upstream TSS of human, and overlies the EWS motif identified in human and mouse [30]. Hence, the CAGE data we have generated is consistent with accurate location of TSS in a complex promoter.

### Validation by RNA-Seq

RNA-Seq was carried out on pig macrophage libraries (see methods) from alveolar macrophages (AM) and bone marrow-derived macrophages (BMDM), in presence or absence (control – CTRL) of lipopolysaccharide (LPS). The primary sequence data were previously provided to the pig genome consortium and contributed to annotation (Pig Genome and Immunome papers), but have not otherwise been analysed. Each RNA-Seq paired-end library was aligned to the pig genome (repeat-masked) with Tophat2. The aligned reads were then assembled into transcripts with Cufflinks. All these assemblies were then merged with Cuffmerge. The number of fragments uniquely mapped to each known transcript was calculated using HTSeq-count [33] and normalized by library size and gene length with counts converted to fragments per kilobase of exon per million fragments mapped (FPKM) [34]. These macrophage RNA-Seq libraries show expression of 14,186 known annotated pig genes (FPKM>0) with gene expression reported in at least one of the libraries – of which 10,832 were expressed at FPKM>1.

We compared the locations of human FANTOM5 promoters mapped to the pig genome with pig RNA-Seq expression signal to identify those mapped promoters with evidence of expression in pig macrophage cells. In this process, all mapped reads were included in order to account for the qualitative expression of genes within duplicated gene families. The midpoint locations were first extracted from the mapped promoters and extended 5 Kb upstream and 20 Kb downstream with regard to the 5’-to-3’ orientation (*i.e.* strand-dependent extension). All extended genomic regions for all mapped promoters were divided in 100 bp non-overlapping windows. RNA-Seq read counts were reported per genomic window. The coverage across all genomic windows for each of the mapped promoter is presented as heatmaps and only those promoters with a minimum of 11 reads across the first 100 windows (covering the midpoint location at window numbered 52) are included (*i.e.* 114,130 promoters). Heatmaps for all chromosomes (1–18, X, Y, MT) are provided as Additional file 8: Figures S4A-S4U. There is a clear expression signal surrounding most of the mapped promoters on the main chromosomes (incl. 1–18, X, Y) as seen by the increase in coverage in the few genomic windows around the midpoint location. Evidence of nearby expression was assessed based on the RNA-Seq expression near the mapped midpoints from 200 bp upstream (5’-end) to 400 bp downstream (3’-end): a minimum of 1 FPKM expression was required in any of the seven 100 bp non-overlapping windows (including the window containing the midpoint) covering the described genomic range to report evidence of nearby expression. A total of 42,541 (37%) mapped promoters fulfilled this criterion. The remaining promoters lacking a nearby pig RNA-Seq signal are either associated with weak expression profiles or associated with a gene located further away from the actual mapped promoter, or may also correspond to promoters of genes which are not expressed in the cells and conditions sampled.

From 7-8% (AM/LPS and AM/CTRL respectively) to 12% (2 BMDM libraries) of the paired-end reads in each library did not map to the genome. These unmapped reads were pooled, unpaired reads were filtered out and the remaining paired-end reads were subjected to *de novo* assembly. The assembled transcripts were then mapped to the NCBI nucleotide database. Additional file 9: Table S5 shows summary statistics of the mapped assembled transcripts. There were 332 assembled transcripts mapping human-annotated sequences and not mapped to pig sequences (based on the top 5 best hits per assembled transcript query). Although these *de novo* assembled transcripts do not map to pig sequences, they map to human genes that are annotated in the pig and therefore do not support the identification of novel gene models.

Additionally the reads mapped to the pig genome that do not overlap with any known annotations were added to the pool of unmapped reads and aligned to the pig Unigene sequences (50,106 sequences). A total of 22,209 Unigene sequences were expressed (>= 1 FPKM) in at least one of the macrophage libraries – 1,974 (9%) of which were only expressed in LPS treated cells. This number reduces to 1,938 (9%) when removing those Unigene sequences that can now be associated with an Ensembl ID based on the mapping of all the unique sequences in the Snowball array probe sets [35] to the pig genome and overlapping those mapped locations with the latest available set of gene annotations (Ensembl API v77).

## Discussion and conclusions

As discussed in the introduction, the current annotation of the pig transcriptome is relatively sparse compared to mouse and human. Notwithstanding that limitation, the available transcriptomic information enabled the development of a reasonably comprehensive microarray platform, and the development of a preliminary pig gene expression atlas [35]. Meta-analysis of public domain data has produced even more comprehensive transcriptional atlas resources from mouse [36], human [37] based upon comprehensive and well-annotated microarray platforms. Co-expression clustering has provided insights into the likely functions of many protein-coding genes; the principal of guilt-by-association [35]. For example, the clustering of the pig expression data revealed two separate clusters of genes associated with mitochondrial function.

A significant deficiency of microarrays is their limited ability to discriminate variation in the precise transcript expressed. For example, the microphthalmia transcription factor (MITF) has well-documented function in differentiation in several distinct lineages, and has multiple alternative 5’ non-coding exons driven by different promoters [38, 39]. Because of the complex regulation, coexpression analysis based upon microarrays does not link MITF to its known target genes in different cell types. The FANTOM5 consortium produced an even more extensive transcriptional atlas in mice and humans based upon the use of CAGE tag frequencies [9]. The data reveal, for example, that MITF has at least 7 promoters over a 190 Kb genomic region. The most proximal is restricted to melanocytes, thereby linking MITF to its well-known control of genes involved in melanocyte pigment production [38], which is also seen in pigs [40], whereas the most distal promoter is expressed in macrophages. All of the human MITF-associated promoters were mapped to the pig genome. The pig macrophage-derived CAGE data detected all of the promoters with at least one tag, but was mainly associated with one of them as expected (not shown). However, the current pig assembly shows two copies of MITF, and most of the predicted promoters based upon human promoter mapping lie downstream and in the opposite orientation to both copies. Only three promoters are located upstream of one copy of MITF and overlap a lowly expressed pig CAGE-tag starting site cluster (supported by 2 tags) at a similar position –with regard to the human MITF 5’-end- to that of a human FANTOM5 CAGE cluster mostly expressed in melanocytes and weakly in human macrophages. The mapped human promoters could provide long-range information that would support improved assembly of this region.

An unexpected outcome of the FANTOM5 analysis was that CAGE profiling also identified around 40,000 active enhancers in the human genome, based upon their bidirectional promoter activity [8]. Furthermore, both this analysis, and previous data from ENCODE [6] have demonstrated that allelic variants (SNVs) at promoter-enhancer regions are more likely to be connected to phenotype in genome-wide association studies [41]. The conservation of the CAGE-defined promoter activity between mouse and human has been explored previously [42]. However, the publication of the mouse ENCODE [14] revealed substantial divergence of sequences involved in transcriptional regulation, chromatin state and higher order chromatin organization between mice and humans, especially amongst genes involved in immune responses.

The mapping of the FANTOM5 human and mouse promoter /enhancer data to pig genome clearly provides an *a priori* tool to support genome annotation and functional genomics in this important species. Despite the less-complete sequence/assembly of the pig genome, a substantially greater proportion of human promoters and candidate enhancers mapped only to the pig genome than to the mouse genome. Although we looked only at data from a single source, macrophages in various states of activation, around 54% of highly confident human promoter mappings to the pig genome were supported by complementary evidence from pig CAGE (incl. uniquely mapped singletons) and/or pig RNA-Seq data (at most 2 Kb away), of which 25% were supported by both pig CAGE and RNA-Seq data. Accordingly, we suggest that these mapped promoters, together with our previously-published microarray-based resource, provide *a priori* functional annotations based upon expression. The set of human genes (3799) associated with high-confidence mapped FANTOM5 human promoters to the pig genome -based on the additional evidence from CAGE and RNA-Seq expression signal- was further analysed to identify the biological processes in which they were involved. GO enrichment analysis for this set of genes shows the many processes statistically significantly enriched in which those genes play an important role (see Additional file 10: Table S6). Thus, the comparative approach applied in this study could provide useful preliminary annotation of other animal genomes for genes involved in many biological pathways.

As CAGE and other data are accumulated by the FAANG consortium [5], it will be interesting to compare and contrast gene expression and the gain and loss of regulatory elements between pigs and humans. Despite the high level of validation of predictions of TSS from cross-mapping of the human FANTOM5 data, it remained the case that the majority of pig CAGE tags were not coincident with those locations. This set will include predicted enhancers. The depth of sequencing and number of libraries we have used is insufficient to identify those elements reliably based upon CAGE data.

**Fig. 4:**
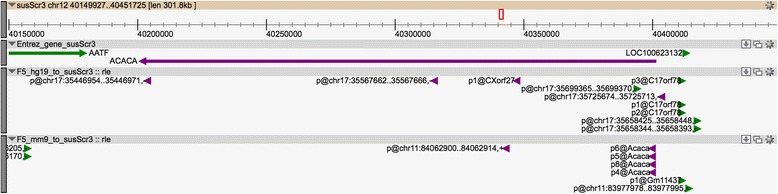
Pig genome browser view of *ACACA* gene locus. Pig genome browser view (Zenbu [55]) of the pig genome at the *ACACA* gene locus with the following tracks displayed: NCBI Entrez gene track, FANTOM5 human and mouse promoter mappings. Features located on forward and reverse strands are displayed in green and violet respectively

**Fig. 5:**
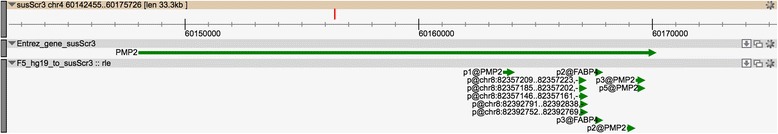
Pig genome browser view of *PMP2* gene locus. Pig genome browser view (Zenbu [55]) of the pig genome at the *PMP2* gene locus with the following tracks displayed: NCBI Entrez gene track, FANTOM5 human promoter mappings. Features located on forward and reverse strands are displayed in green and violet respectively

**Fig. 6:**
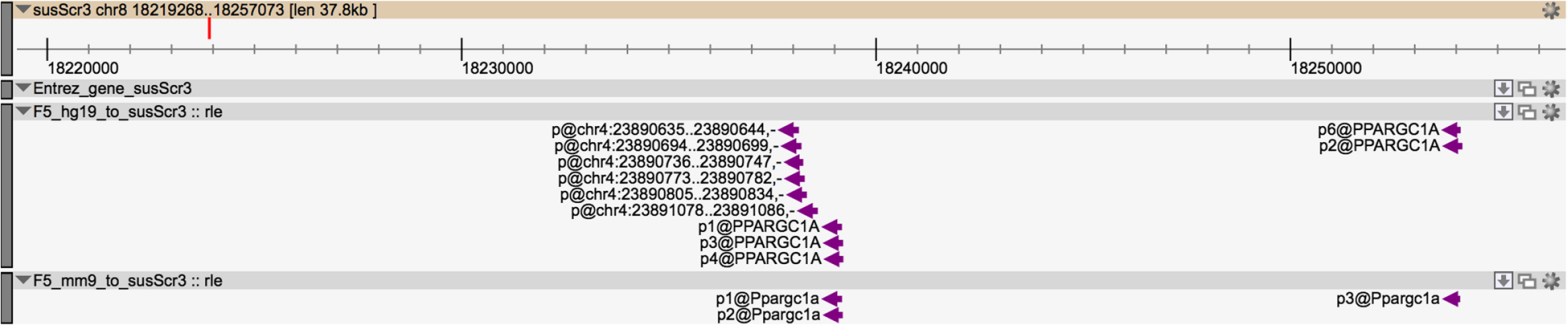
Pig genome browser view of *PPARGC1A* gene locus. Pig genome browser view (Zenbu [55]) of the pig genome at the *PPARGC1A* gene locus with the following tracks displayed: NCBI Entrez gene track, FANTOM5 human and mouse promoter mappings. Features located on forward and reverse strands are displayed in green and violet respectively

Part of the pig genome paper [43] and a satellite paper [44] included detailed annotation of immune-associated genes of the pig. In the course of our own studies, we have identified a number of loci that are either absent, or poorly assembled, in the current build of the genome (*e.g.* the *CSF1R*, *IFITM, MITF* loci) and the pig macrophage CAGE data further supports the view that there are many genes that are absent or incorrectly assembled in the current pig genome assembly. Other studies also reported on various incorrect annotations or non-annotated genes in the current build [23, 45]. For example, in a study looking at muscle transcriptome in pigs, three genes were reported to be incorrectly (*ACACA*, *PPARGC1A*) or non-annotated (*FABP4*) in the current version of the pig genome (Sscrofa10.2). Four FANTOM5 mouse promoters were mapped upstream of the 5’-end of porcine *ACACA* gene as well as one unannotated human promoter (Fig. 4). There was no RNA-Seq signal for this gene in our data but a pig CAGE signal in the middle of the current gene model could be defining a novel alternative transcript. *FABP4* -although not annotated in Ensembl- is associated with an EntrezGene ID but none of the human/mouse promoters were mapped in its vicinity (nor within the annotated coding sequence). Two of the three human FANTOM5 promoters associated with the human *FABP4* gene were mapped in the coding region of the pig *PMP2*. All the mapped human promoters of the *PMP2* gene also map within the pig *PMP2* locus (Fig. 5). This observation confirms the incorrect annotation of *PMP2* and *FABP4* loci in the pig genome and might help in the identification of the correct porcine gene locations. The pig *PPARGC1A* gene is annotated on an unplaced scaffold (GL896199.1), its 5’-end is located near the end of the scaffold on the reverse strand which prevents the localization of upstream promoters for this locus. The FANTOM5 datasets suggest a different location for this gene with two distinct clusters of promoters each supported by FANTOM5 promoters from both human and mouse datasets (Fig. 6).

Although the pig is clearly economically important in its own right, part of the motivation for systematic annotation is to support the use in biomedical research. Genome sequencing (pig genome), as well as recently-enabled genome-wide exome sequencing [46] reveals that, like humans, each pig genome contains large numbers of potentially deleterious variant as well as clear loss-of-function mutations. The advantage of the pig is that it is multiparous, so that such variants can be potentially crossed to homozygosity through brother-sister mating. A large metaanalysis by Seok *et al.* [47] cast doubt upon the value of the mouse as a model for human inflammatory diseases. A subsequent analysis of the same data concluded that the correlation was somewhat better, but only if the focus was restricted to the set of genes that is inducible in both species [48]. As we noted previously, there is a large set of genes that is completely discordantly regulated in mouse and human macrophages responding to lipopolysaccharide (LPS) [3] and pigs are much more human-like in this response [12]. The current study supports the use of the pig as a model for human disease [2], and also provides resources for analysis of quantitative traits and single gene effects in this species.

## Dataset and methods

### FANTOM5 CAGE data and promoters

The large collection of human and mouse Cap Analysis of Gene Expression (CAGE) libraries was generated as part of the FANTOM5 consortium effort [9].

Human and mouse promoters were defined as 501 bp genomic windows including 400 nucleotides upstream and 100 downstream of the main CAGE-defined TSS (CAGE-derived promoter region referred to as [−400, TSS, +100]). The human promoters were aligned to the pig and mouse genomes (Sscrofa10.2 and GRCm37/mm9, respectively), and the mouse promoters were mapped to the pig and human genomes (Sscrofa10.2 and GRCh37/hg19, respectively). Alignments of FANTOM5 CAGE-derived promoters to a target genome were performed using blastn (NCBI blast-2.2.25+) in discontiguous megablast mode. The Additional file 1: Figure S1 describes the FANTOM5 promoter comparative analysis framework. The FANTOM5-defined promoters were first mapped to a set of known orthologous regions (upstream regions of orthologous genes as defined below) between the genes in the genome the FANTOM5 CAGE tags were derived from and genes in the target genome considered. In order to define the sets of orthologous regions, the lists of porcine genes orthologous to human (mouse) genes were extracted using Ensembl Biomart (release 67) as well as human-to-mouse orthologs. The upstream regions of each orthologous gene were defined as genomic windows of 2101 bases containing 2 Kb upstream and 100 bases downstream of the gene 5’-end position.

CAGE-derived human promoters mapping to at least one of the known orthologous regions are reported as mapped to the target species. All human promoters not mapped in the first step (*i.e.* orthology mapping) were mapped to the repeat-masked target genome and only those promoters mapped uniquely to the target genome were reported. Additionally the multimapped promoters were further rescued based on the bit-score ranking of all hits for each promoter and selecting the top hit if the score ratio between the second best hit and the top hit was below 0.95. For those multimapped promoters failing this score ratio criteria, one of the two top hits was considered a single hit whenever it was located on a chromosome and all other hits were located on unplaced scaffolds.

Alignment of promoters to the repeat-masked target genome was performed using blastn with discontiguous megablast mode from the NCBI blast-2.2.25+ suite of tools with the following parameters: −task dc-megablast, penalty (−1), reward (1), gapopen (0), gapextend (2), perc_identity (20), evalue (0.01) and outfmt (5). The discontiguous megablast mode was used to allow for the comparison of diverged sequences from distinct organisms (cf. BLAST+ user manual at http://www.ncbi.nlm.nih.gov/books/NBK279690/). The pig genome (Sscrofa10.2) was repeat-masked using RepeatMasker (version open-3.3.0) with the following parameters: engine (ncbi), species (“Sus scrofa”), pa (8), s (−no_is), cutoff (255) and frag (20000). NCBI makeblastdb (version 2.2.25+) was run on all upstream windows for each target genome and on the pig repeat-masked genome to get the blast-formatted databases.

The unmapped promoters were then re-mapped to the repeat-masked target genome using blastn with the following modified parameters: −penalty −3 -reward 1 -gapopen 3 -gapextend 3. Promoters were considered single mappers if they were uniquely mapped. Multimapped promoters were rescued in the same way as that previously described for the mapped promoters in the first iteration.

Mouse promoters were derived from FANTOM5 CAGE data in the same way as that described for the human promoters and were mapped to the repeat-masked mouse genome (mm9) as downloaded from Ensembl (API 67).

#### Protein-coding genes with FANTOM5 promoter prediction in pig

The set of non-redundant Ensembl IDs that were mapped to the pig genome (based on the mapping of FANTOM5 human and mouse promoters) covered 16,828 genes which correspond to (i) the union of all mouse Ensembl IDs from the non-redundant list that can be mapped to pig -using human-to-mouse orthologs to convert all human Ensembl IDs (from 15,797 human to 13,856 mouse Ensembl IDs) and an additional 1,031 mouse Ensembl IDs from the mouse promoter mappings- which leads to 14,887 Ensembl IDs and (ii) the 1,941 human Ensembl IDs from the non-redundant list for which there is no known mouse ortholog (*i.e.* 15,797-13,856).

### Pig CAGE

A pig CAGE library was prepared from alveolar macrophages as described in [49] from one female Large White x Landrace animal (about 9 weeks old) and sequenced on an Illumina GA platform. This resulted in a library of 3.9 M (3,899,341) 36 bp single-end reads. Raw reads were trimmed from adapter sequence at their 3’ end, and trimmed at 5’ end to remove barcode and restriction enzyme sequences with cutadapt (v1.0). Trimmed reads were then filtered using sickle (single end mode) to keep only those reads with a minimum length of 20 bases. The resulting library was aligned to the pig genome using Novoalign (v2.07.18) with soft clipping at both ends by at most two bases (−s2). The unmapped reads (39%) were further trimmed from their 5’-end and 3’-end by one base and realigned iteratively until either a single mapping location was identified or the trimmed read reached a minimum length threshold (11bp) at which point the iteration was stopped. The length threshold was selected based on the shortest unique substring observed in human and mouse genomes which is of length 11 bp [50]. A single mapping location was reported whenever a read was uniquely mapped or based on the score ratios between the top two best hits -the top hit was considered to be uniquely mapped if the score ratio (s2/s1) between the second hit (s2) and the first (s1) was below 0.95. Failing the score ratio criteria, one of the two top hits was considered a single hit whenever it was located on a chromosome and all other hits were located on unplaced scaffolds.

### Pig RNA-Seq

RNA-Seq libraries were prepared from a male Large White x Landrace pig (about 7 weeks old). Unstranded 35 bp paired-end RNA-Seq libraries from alveolar macrophages (AM) were generated following the protocol described in [51]. Unstranded 57 bp paired-end RNA-Seq libraries from bone-marrow-derived macrophages (BMDM) were generated following the protocol described in [52]. In total, four macrophage libraries were generated - two per tissue: two controls (untreated AM and BMDM samples) and two samples were additionally treated with LPS (referred to as AM/LPS and BMDM/LPS respectively).

Macrophage RNA-Seq paired-end reads were trimmed using sickle (v1.1) -keeping reads with base quality of at least Q20 and a minimum read length after trimming of 20 bp- and aligned to the pig genome (repeat-masked) with TopHat2/Bowtie2. The pig genome was repeat-masked in order to restrict the genomic search space and extract a set of high-confidence mapping fragments. Library-specific insert sizes were estimated by mapping the trimmed reads to the pig transcriptome. The unmapped read pairs were assembled into transcripts using SOAPdenovo-Trans-31kmer (version 1.02) and aligned to the NCBI nucleotide database (version as of 2012-09-12).

### Animal ethics

FANTOM5 human and mouse samples: please refer to the methods section in the supplementary information from [9].

Pig CAGE and RNA-Seq samples: all the pigs spent at least 2 weeks in the same facility at rest before experimentation. Animals have not shown any signs of any infections, did not receive any vaccinations and the female pig was not pregnant. All animal studies were conducted according to University of Edinburgh Guidelines and were approved by the Institutional Ethics Committee [52].

### Target genomes in cross-species vs. within species promoter mappings

In cross-species alignments, a repeat-masked target genome was used in order to get a high-confidence set of FANTOM5 (human or mouse) promoters mapped unequivocally to the target genome. In the case of within-species promoter mappings, pig CAGE data were aligned to the whole unmasked pig genome, which led to additional evidence for pig retro-transposon transcription.

### Genomic feature proximity

To compare the “overlap” between the cross-species CAGE promoter mappings and the pig-specific CAGE mappings to the pig genome, features were considered to be in proximity if they were distant by at most 2 Kb.

In RNA-Seq read coverage analysis, the window size was set to 100 bp to analyse the read coverage in small genomic regions to allow for the detection of transition in read coverage around the mapped promoters and beyond. The RNA-Seq read coverage was looked at across a wide genomic window (5 Kb upstream to 20 Kb downstream of the mapped promoter mid-point location) to further assess the proximity of the mapped FANTOM5 promoters to the observed RNA-Seq expression signal in the pig genome.

The BEDTools [53] suite of tools was used to identify the closest genes to mapped features and define overlaps between features.

## Availability of supporting data

The FANTOM5 human and mouse CAGE-derived promoters mapped to the pig genome (version 10.2) are provided to the users as a track hub (http://pubdata.roslin.ed.ac.uk/genomics/farm-animal/susScr3/SuppData/hub.txt) as well as the mappings of pig CAGE CTSS and RNA-Seq datasets generated for this study to visualize in a genome browser. BED files for the FANTOM5 human/mouse mappings to the pig genome are also available as Additional file 11: Table S7 and Additional file 12: Table S8 respectively. The pig CAGE CTSS mappings are available in Additional file 13: Table S9 and Additional file 14: Table S10 for uniquely mapped and multimapped tags respectively.

The datasets supporting the results of this article are available in the European Nucleotide Archive repository, PRJEB10004, http://www.ebi.ac.uk/ena/data/view/PRJEB10004.

## Additional files

Additional file 1: Figure S1. FANTOM5 promoter comparative analysis framework. FANTOM5 promoters were extended from 400 bases upstream to 100 bases downstream of their main TSS. Orthologous genomic regions were extracted for all genes within the target genome –orthologs between the species the FANTOM5 promoters belong to (human/mouse) and that of the target genome (pig/human/mouse). These regions were extracted as 2.1 Kb windows containing 2 Kb upstream and 100 bp downstream from each orthologous genes’ 5’-end. Promoters mapping to at least one known orthologous region were reported with their mapping locations, while the remaining set of promoters were mapped to the whole target genome (repeat-masked). The set of uniquely mapped promoters is reported with their genomic locations. The multimapped promoters were filtered based on the score ratio of the top two best hits -the top hit was considered to be uniquely mapped if the score ratio (s2/s1) between the second hit (s2) and the first (s1) was below 0.95. Failing the score ratio criteria, one of the two top hits was considered a single hit whenever it was located on a chromosome and all other hits were located on unplaced scaffolds. The unmapped promoters were re-aligned (2^nd^ run - see methods) and the same procedure was followed to report the uniquely mapped promoters. Two sets of genes were extracted from the final set of unmapped FANTOM5 human promoters to the pig genome for GO terms enrichment analysis (see text): the set of genes with at least one FANTOM5 promoter unmapped (referred to as genes_tss) and the set of genes with all associated FANTOM5 promoters unmapped (referred to as genes_none). (PDF 259 kb) Additional file 2: Table S2. Table S2A – Locations of FANTOM5 human promoters mapped to the pig genome. Table contains the following columns (left to right): FANTOM5 human promoter ID, Ensembl gene ID associated with the FANTOM5 human promoter, mapping locations to the pig genome (Sscrofa10.2), the nearest pig Ensembl ID to the mapped promoter (strand-specific) and the number of pig CAGE CTSS and/or RNA-Seq signal (assembled transcripts from pooled macrophage libraries) located within a 20 Kb window from the 3’-end of the mapped promoter. Table S2B – Locations of FANTOM5 human promoters mapped to the mouse genome. Table contains the following columns (left to right): FANTOM5 human promoter ID, Ensembl gene ID associated with the FANTOM5 human promoter, mapping locations to the mouse genome (mm9), the nearest mouse Ensembl ID to the mapped promoter (strand-specific). (ZIP 5853 kb) Additional file 3: Table S1. GO enrichment analysis for the human genes with FANTOM5 human TSS unmapped to the pig genome. Table shows the list of GO terms enriched for two sets of human genes: (i) for which at least one FANTOM5 human TSS was not mapped to the pig genome (class referred to as genes_tss) and (ii) for which all the associated FANTOM5 human TSS were not mapped to the pig genome (class referred to as genes_none). GO enrichment analysis was performed against a background set consisting of all the human genes with a known associated FANTOM5 human TSS using GOrilla [54]. The columns from left to right indicate: GO terms ID; description of the GO term; P-value; FDR adjusted q-value (*i.e.* P-value corrected for multiple testing using the Benjamini and Hochberg’s method); Enrichment; total number of genes (N); total number of genes associated with a specific GO term (B); number of genes in the target set (n); number of genes in the intersection (b). Enrichment is defined as (b/n)/(B/N) [54]. The last column (Genes) displays the list of associated genes that appear in the optimal top of the list – each gene name is shown with its gene symbol followed by a short description of the gene. (XLS 1049 kb) Additional file 4: Table S3. Table S3A: Locations of FANTOM5 mouse promoters mapped to the pig genome. Table contains the following columns (left to right): FANTOM5 mouse promoter ID, Ensembl gene ID associated with the FANTOM5 mouse promoter, mapping locations to the pig genome (Sscrofa10.2), the nearest pig Ensembl ID to the mapped promoter (strand-specific) and the number of pig CAGE CTSS and/or RNA-Seq signal (assembled transcripts from pooled macrophage libraries) located within a 20 Kb window from the 3’-end of the mapped promoter. Table S3B: Locations of FANTOM5 mouse promoters mapped to the human genome. Table contains the following columns (left to right): FANTOM5 mouse promoter ID, Ensembl gene ID associated with the FANTOM5 mouse promoter, mapping locations to the human genome (hg19), the nearest human Ensembl ID to the mapped promoter (strand-specific). (ZIP 2981 kb) Additional file 5: Figure S2. Distribution of FANTOM5 human promoters’ expression – mappings proximal or distant from pig CAGE CTSS clusters. **S2A**: FANTOM5 human promoter mapped with proximal (<=2 Kb) pig CAGE CTSS cluster. **S2B**: FANTOM5 human promoter mapped without a nearby pig CAGE CTSS cluster (>2 Kb). The y-axis shows the FPKM expression values; the x-axis shows the number corresponding to each of the 55 FANTOM5 monocyte and macrophage libraries as described in the Additional file 6: Table S4. (ZIP 282 kb) Additional file 6: Table S4. Description of the FANTOM5 monocyte and macrophage libraries. Table contains a summary description for each of the 55 FANTOM5 monocyte and macrophage libraries - as provided by the FANTOM5 consortium. (CSV 5 kb) Additional file 7: Figure S3. Distribution of tags per pig CAGE CTSS cluster with/without proximal mapped FANTOM5 human promoter. Boxplots of the number of tags per pig CAGE CTSS cluster with a proximal mapped FANTOM5 human promoter –at most 2 Kb away- (left) and without a nearby mapped FANTOM5 human promoter (right). (PDF 167 kb) Additional file 8: Figures S4A-S4U. Heatmaps of pig RNA-Seq coverage across FANTOM5 human promoters mapped to the pig genome. Heatmaps showing the RNA-Seq coverage across successive 100 bp genomic windows spanning 5 Kb upstream and 20 Kb downstream of each mapped FANTOM5 human promoter (midpoint of the mapped region taken as reference). FANTOM5 promoter IDs are stacked on the y-axis (labels not displayed). The x-axis corresponds to the successive 100 bp genomic windows, with the window numbered 52 corresponding to the promoter midpoint region. Only those promoters with a minimum of 11 reads across the first 100 windows (covering the midpoint location) were included (*i.e.* 114,130 promoters). Figures S3A to S3U correspond to heatmaps for the porcine chromosomes: 1–18, X, Y and MT respectively. (ZIP 78361 kb) Additional file 9: Table S5. Assembled transcripts mapping statistics to the NCBI nucleotide database based on the originally unmapped (to Sscrofa10.2) RNA-Seq fragments. RNA-Seq unmapped fragments from four macrophage libraries were pooled, assembled as transcripts using SOAPdenovo-Trans-31kmer and aligned to the NCBI nucleotide database (NT). Statistics show the number of transcripts assembled, mapped to NT, and expressed at FPKM>0 or FPKM>10 with pig or human annotation present in at least one hit of the top five hits to NT. (XLSX 26 kb) Additional file 10: Table S6. List of enriched GO terms for the set of human genes associated with high-confidence mapped FANTOM5 human promoters to the pig genome. List of enriched GO terms for the target set of 3794 human genes which are associated with FANTOM5 human promoters mapped with high confidence to the pig genome (*i.e.* supported by pig CAGE and RNA-Seq expression signal) versus the background set of human genes with FANTOM5 human promoter. GO enrichment analysis was performed using GOrilla [54]. See Additional file 3: Table S1 for the description of each column. (XLS 4622 kb) Additional file 11: Table S7. Mapping locations of FANTOM5 human promoters to the pig genome (BED). Table (BED6 format) contains the following columns (left to right): chromosome, start, end, FANTOM5 human promoter ID, colour code and strand. The first three and sixth columns refer to the mapped locations of FANTOM5 human promoters (as identified by their ID in column 4) onto the pig genome. (TSV 6432 kb) Additional file 12: Table S8. Mapping locations of FANTOM5 mouse promoters to the pig genome (BED). Table (BED6 format) contains the following columns (left to right): chromosome, start, end, FANTOM5 mouse promoter ID, colour code and strand. The first three and sixth columns refer to the mapped locations of FANTOM5 mouse promoters (as identified by their ID in column 4) onto the pig genome. (TSV 3015 kb) Additional file 13: Table S9. Mapping locations of the pig CAGE CTSS to the pig genome: uniquely mapped subset (BED). Table contains the following columns (left to right): chromosome, start, end, pig CAGE CTSS ID (strand-specific), number of tags in the CTSS and strand. The first three and sixth columns refer to the mapped locations of the pig CAGE CTSS (as identified by the ID in column 4) onto the pig genome. The set of pig CAGE CTSS reported includes only the uniquely mapped pig CAGE tags. (TSV 3840 kb) Additional file 14: Table S10. Mapping locations of the pig CAGE CTSS to the pig genome: multimapped subset (BED). Table contains the following columns (left to right): chromosome, start, end, pig CAGE CTSS ID (strand-specific), number of tags in the CTSS and strand. The first three and sixth columns refer to the mapped locations of the pig CAGE CTSS (as identified by the ID in column 4) onto the pig genome. The set of pig CAGE CTSS reported includes only the multimapped pig CAGE tags. (TSV 5734 kb)

## Abbreviations

AM: Alveolar macrophages
BMDM: Bone marrow-derived macrophages
CAGE: Cap analysis of gene expression
ChIP-Seq: Chromatin immunoprecipitation sequencing
CNV: Copy number variant
CTRL: Control
CTSS: CAGE-tag starting site cluster
ENCODE: Encyclopedia of DNA elements
eRNA: Enhancer RNA
FAANG: Functional annotation of animal genomes
FANTOM5: Functional annotation of mammalian genomes 5
FDR: False discovery rate
FPKM: Fragments per kilobase of exon per million fragments mapped
GO: Gene ontology
HS: DNaseI-hypersensitive
LINE: Long interspersed nuclear element
LPS: Lipopolysaccharide
LTR: Long terminal repeat
RNA-Seq: RNA sequencing
SINE: Short interspersed nuclear element
SNV: Single nucleotide variant
TE: Transposable element
TLS: Translation start site
TSS: Transcription start site

## References

[1] Knox RV (2014). Impact of swine reproductive technologies on pig and global food production. Adv Exp Med Biol.

[2] Meurens F, Summerfield A, Nauwynck H, Saif L, Gerdts V (2012). The pig: a model for human infectious diseases. Trends Microbiol.

[3] Fairbairn L, Kapetanovic R, Sester DP, Hume DA (2011). The mononuclear phagocyte system of the pig as a model for understanding human innate immunity and disease. J Leukoc Biol.

[4] Groenen MAM, Archibald AL, Uenishi H, Tuggle CK, Takeuchi Y, Rothschild MF (2012). Analyses of pig genomes provide insight into porcine demography and evolution. Nature.

[5] Andersson L, Archibald AL, Bottema CD, Brauning R, Burgess SC, Burt DW (2015). Coordinated international action to accelerate genome-to-phenome with FAANG, the Functional Annotation of Animal Genomes project. Genome Biol.

[6] Dunham I, Kundaje A, Aldred SF, Collins PJ, Davis CA, Doyle F (2012). An integrated encyclopedia of DNA elements in the human genome. Nature.

[7] Yue F, Cheng Y, Breschi A, Vierstra J, Wu W, Ryba T (2014). A comparative encyclopedia of DNA elements in the mouse genome. Nature.

[8] Andersson R, Gebhard C, Miguel-Escalada I, Hoof I, Bornholdt J, Boyd M (2014). An atlas of active enhancers across human cell types and tissues. Nature.

[9] The FANTOM Consortium and the RIKEN PMI and CLST (DGT)- (2014). A promoter-level mammalian expression atlas. Nature.

[10] Wernersson R, Schierup MH, Jørgensen FG, Gorodkin J, Panitz F, Staerfeldt H-H (2005). Pigs in sequence space: a 0.66X coverage pig genome survey based on shotgun sequencing. BMC Genomics.

[11] Rye M, Sandve GK, Daub CO, Kawaji H, Carninci P, Forrest ARR (2014). Chromatin states reveal functional associations for globally defined transcription start sites in four human cell lines. BMC Genomics.

[12] Kapetanovic R, Fairbairn L, Beraldi D, Sester DP, Archibald AL, Tuggle CK (2012). Pig bone marrow-derived macrophages resemble human macrophages in their response to bacterial lipopolysaccharide. J Immunol.

[13] Wernersson R, Schierup MH, Jørgensen FG, Gorodkin J, Panitz F, Staerfeldt H-H (2005). Pigs in sequence space: a 0.66X coverage pig genome survey based on shotgun sequencing. BMC Genomics.

[14] Yue F, Cheng Y, Breschi A, Vierstra J, Wu W, Ryba T (2014). A comparative encyclopedia of DNA elements in the mouse genome. Nature.

[15] Faulkner GJ, Forrest AR, Chalk AM, Schroder K, Hayashizaki Y, Carninci P (2008). A rescue strategy for multimapping short sequence tags refines surveys of transcriptional activity by CAGE. Genomics.

[16] Feschotte C (2008). Transposable elements and the evolution of regulatory networks. Nat Rev Genet.

[17] Mariño-Ramírez L, Jordan IK (2006). Transposable element derived DNaseI-hypersensitive sites in the human genome. Biol Direct.

[18] Dong K, Pu Y, Yao N, Shu G, Liu X, He X, et al. Copy number variation detection using SNP genotyping arrays in three Chinese pig breeds. Anim Genet. 2015. 10.1111/age.12247 25590996

[19] Paudel Y, Madsen O, Megens H-J, Frantz LAF, Bosse M, Bastiaansen JWM (2013). Evolutionary dynamics of copy number variation in pig genomes in the context of adaptation and domestication. BMC Genomics.

[20] Perry GH, Yang F, Marques-Bonet T, Murphy C, Fitzgerald T, Lee AS (2008). Copy number variation and evolution in humans and chimpanzees. Genome Res.

[21] Redon R, Ishikawa S, Fitch KR, Feuk L, Perry GH, Andrews TD (2006). Global variation in copy number in the human genome. Nature.

[22] Pang AW, MacDonald JR, Pinto D, Wei J, Rafiq MA, Conrad DF (2010). Towards a comprehensive structural variation map of an individual human genome. Genome Biol.

[23] Anthon C, Tafer H, Havgaard JH, Thomsen B, Hedegaard J, Seemann SE (2014). Structured RNAs and synteny regions in the pig genome. BMC Genomics.

[24] Wells CA, Ravasi T, Sultana R, Yagi K, Carninci P, Bono H (2003). Continued discovery of transcriptional units expressed in cells of the mouse mononuclear phagocyte lineage. Genome Res.

[25] Massingham T, Goldman N (2012). All Your Base: a fast and accurate probabilistic approach to base calling. Genome Biol.

[26] Dohm JC, Lottaz C, Borodina T, Himmelbauer H (2008). Substantial biases in ultra-short read data sets from high-throughput DNA sequencing. Nucleic Acids Res.

[27] Carninci P, Kasukawa T, Katayama S, Gough J, Frith MC, Maeda N (2005). The transcriptional landscape of the mammalian genome. Science (80-).

[28] Carninci P, Sandelin A, Lenhard B, Katayama S, Shimokawa K, Ponjavic J (2006). Genome-wide analysis of mammalian promoter architecture and evolution. Nat Genet.

[29] Schroder K, Irvine KM, Taylor MS, Bokil NJ, Le Cao K-A, Masterman K-A (2012). Conservation and divergence in Toll-like receptor 4-regulated gene expression in primary human versus mouse macrophages. Proc Natl Acad Sci U S A.

[30] Hume DA, Sasmono T, Himes SR, Sharma SM, Bronisz A, Constantin M (2008). The Ewing sarcoma protein (EWS) binds directly to the proximal elements of the macrophage-specific promoter of the CSF-1 receptor (csf1r) gene. J Immunol.

[31] Ross IL, Yue X, Ostrowski MC, Hume DA (1998). Interaction between PU.1 and another Ets family transcription factor promotes macrophage-specific Basal transcription initiation. J Biol Chem.

[32] Sasmono RT, Oceandy D, Pollard JW, Tong W, Pavli P, Wainwright BJ (2003). A macrophage colony-stimulating factor receptor-green fluorescent protein transgene is expressed throughout the mononuclear phagocyte system of the mouse. Blood.

[33] HTSeq: Analysing high-throughput sequencing data with Python [http://www-huber.embl.de/users/anders/HTSeq/] 10.1093/bioinformatics/btac166 PMC9113351 35561197

[34] Mortazavi A, Williams BA, McCue K, Schaeffer L, Wold B (2008). Mapping and quantifying mammalian transcriptomes by RNA-Seq. Nat Methods.

[35] Freeman TC, Ivens A, Baillie JK, Beraldi D, Barnett MW, Dorward D (2012). A gene expression atlas of the domestic pig. BMC Biol.

[36] Armit C, Venkataraman S, Richardson L, Stevenson P, Moss J, Graham L (2012). eMouseAtlas, EMAGE, and the spatial dimension of the transcriptome. Mamm Genome.

[37] Mabbott NA, Baillie JK, Brown H, Freeman TC, Hume DA (2013). An expression atlas of human primary cells: inference of gene function from coexpression networks. BMC Genomics.

[38] Shibahara S, Takeda K, Yasumoto K, Udono T, Watanabe K, Saito H, Takahashi K: Min. J Investig Dermatol Symp Proc 2001, 6:99–104 10.1046/j.0022-202x.2001.00010.x 11764295

[39] Li M, Zhu F, Hong N, Zhang L, Hong Y (2014). Alternative transcription generates multiple Mitf isoforms with different expression patterns and activities in medaka. Pigment Cell Melanoma Res.

[40] Wang C, Wang H, Zhang Y, Tang Z, Li K, Liu B (2015). Genome-wide analysis reveals artificial selection on coat colour and reproductive traits in Chinese domestic pigs. Mol Ecol Resour.

[41] Chen C-Y, Chang I-S, Hsiung CA, Wasserman WW (2014). On the identification of potential regulatory variants within genome wide association candidate SNP sets. BMC Med Genomics.

[42] Gustincich S, Sandelin A, Plessy C, Katayama S, Simone R, Lazarevic D (2006). The complexity of the mammalian transcriptome. J Physiol.

[43] Archibald AL, Bolund L, Churcher C, Fredholm M, Groenen MAM, Harlizius B (2010). Pig genome sequence--analysis and publication strategy. BMC Genomics.

[44] Dawson HD, Loveland JE, Pascal G, Gilbert JGR, Uenishi H, Mann KM (2013). Structural and functional annotation of the porcine immunome. BMC Genomics.

[45] Puig-Oliveras A, Ramayo-Caldas Y, Corominas J, Estellé J, Pérez-Montarelo D, Hudson NJ (2014). Differences in Muscle Transcriptome among Pigs Phenotypically Extreme for Fatty Acid Composition. PLoS One.

[46] Robert C, Fuentes-Utrilla P, Troup K, Loecherbach J, Turner F, Talbot R (2014). Design and development of exome capture sequencing for the domestic pig (Sus scrofa). BMC Genomics.

[47] Seok J, Warren HS, Cuenca AG, Mindrinos MN, Baker HV, Xu W (2013). Genomic responses in mouse models poorly mimic human inflammatory diseases. Proc Natl Acad Sci U S A.

[48] Takao K, Miyakawa T: Genomic responses in mouse models greatly mimic human inflammatory diseases. Proc Natl Acad Sci 2014 10.1073/pnas.1401965111 PMC4313832 25092317

[49] Kodzius R, Kojima M, Nishiyori H, Nakamura M, Fukuda S, Tagami M (2006). CAGE: cap analysis of gene expression. Nat Methods.

[50] Haubold B, Pierstorff N, Moller F, Wiehe T (2005). Genome comparison without alignment using shortest unique substrings. BMC Bioinformatics.

[51] Endale Ahanda M-L, Fritz ER, Estellé J, Hu Z-L, Madsen O, Groenen MAM (2012). Prediction of altered 3’- UTR miRNA-binding sites from RNA-Seq data: the swine leukocyte antigen complex (SLA) as a model region. PLoS One.

[52] Kapetanovic R, Fairbairn L, Downing A, Beraldi D, Sester DP, Freeman TC (2013). The impact of breed and tissue compartment on the response of pig macrophages to lipopolysaccharide. BMC Genomics.

[53] Quinlan AR, Hall IM: The BEDTools manual. 2010

[54] Eden E, Navon R, Steinfeld I, Lipson D, Yakhini Z (2009). GOrilla: a tool for discovery and visualization of enriched GO terms in ranked gene lists. BMC Bioinformatics.

[55] Severin J, Lizio M, Harshbarger J, Kawaji H, Daub CO, Hayashizaki Y (2014). Interactive visualization and analysis of large-scale sequencing datasets using ZENBU. Nat Biotechnol.

