# Supplementary figures and images for: Identification and annotation of conserved promoters and macrophage-expressed genes in the pig genome
Source: BMC Genomics. 2015 Nov 18;16:970. doi: 10.1186/s12864-015-2111-2 (PMC4652390; doi:10.1186/s12864-015-2111-2)

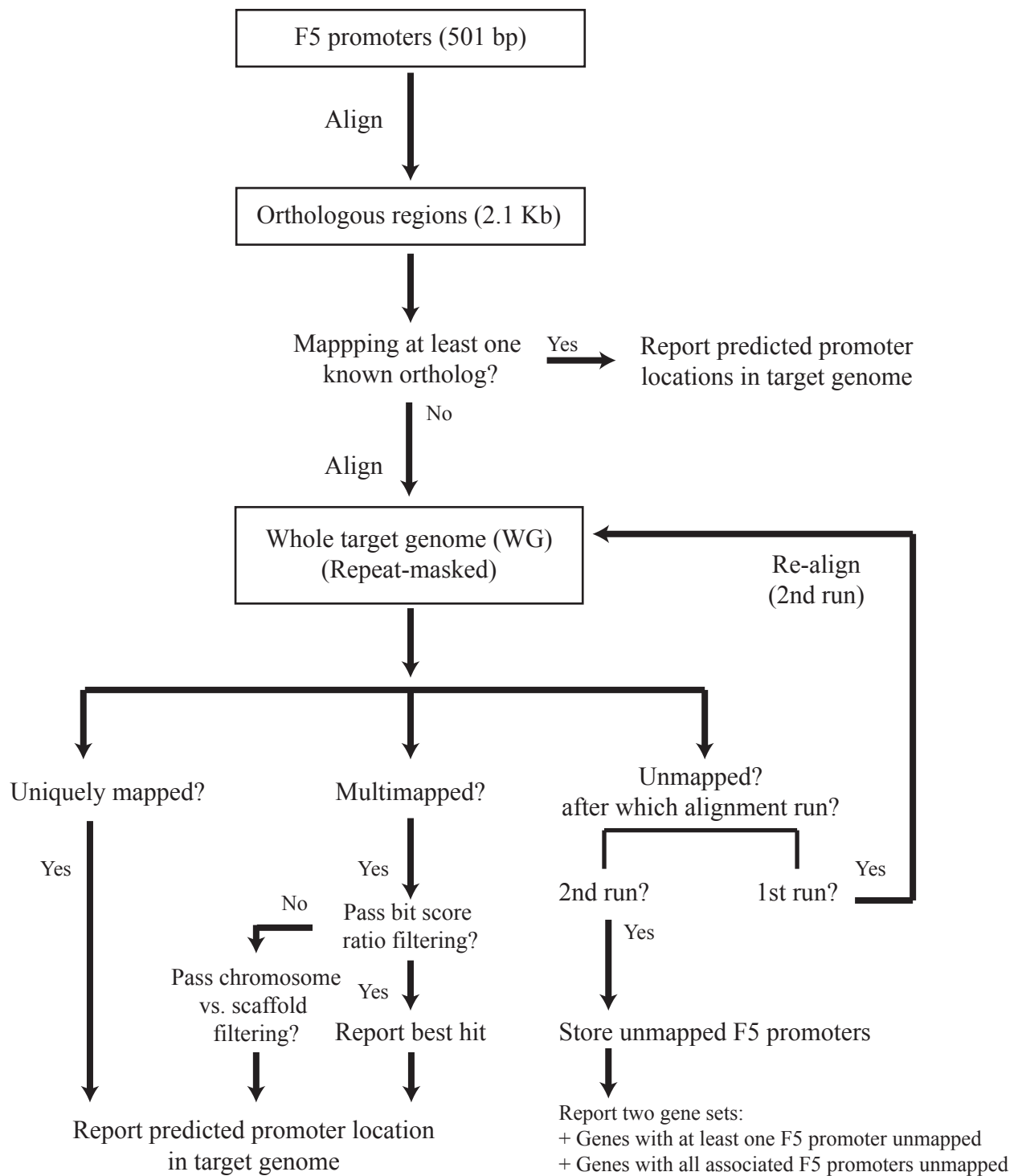

Supplement: Additional file 1: Figure S1. — FANTOM5 promoter comparative analysis framework. FANTOM5 promoters were extended from 400 bases upstream to 100 bases downstream of their main TSS. Orthologous genomic regions were extracted for all genes within the target genome –orthologs between the species the FANTOM5 promoters belong to (human/mouse) and that of the target genome (pig/human/mouse). These regions were extracted as 2.1 Kb windows containing 2 Kb upstream and 100 bp downstream from each orthologous genes’ 5’-end. Promoters mapping to at least one known orthologous region were reported with their mapping locations, while the remaining set of promoters were mapped to the whole target genome (repeat-masked). The set of uniquely mapped promoters is reported with their genomic locations. The multimapped promoters were filtered based on the score ratio of the top two best hits -the top hit was considered to be uniquely mapped if the score ratio (s2/s1) between the second hit (s2) and the first (s1) was below 0.95. Failing the score ratio criteria, one of the two top hits was considered a single hit whenever it was located on a chromosome and all other hits were located on unplaced scaffolds. The unmapped promoters were re-aligned (2nd run - see methods) and the same procedure was followed to report the uniquely mapped promoters. Two sets of genes were extracted from the final set of unmapped FANTOM5 human promoters to the pig genome for GO terms enrichment analysis (see text): the set of genes with at least one FANTOM5 promoter unmapped (referred to as genes_tss) and the set of genes with all associated FANTOM5 promoters unmapped (referred to as genes_none). (PDF 259 kb) [file 12864_2015_2111_MOESM1_ESM.pdf]

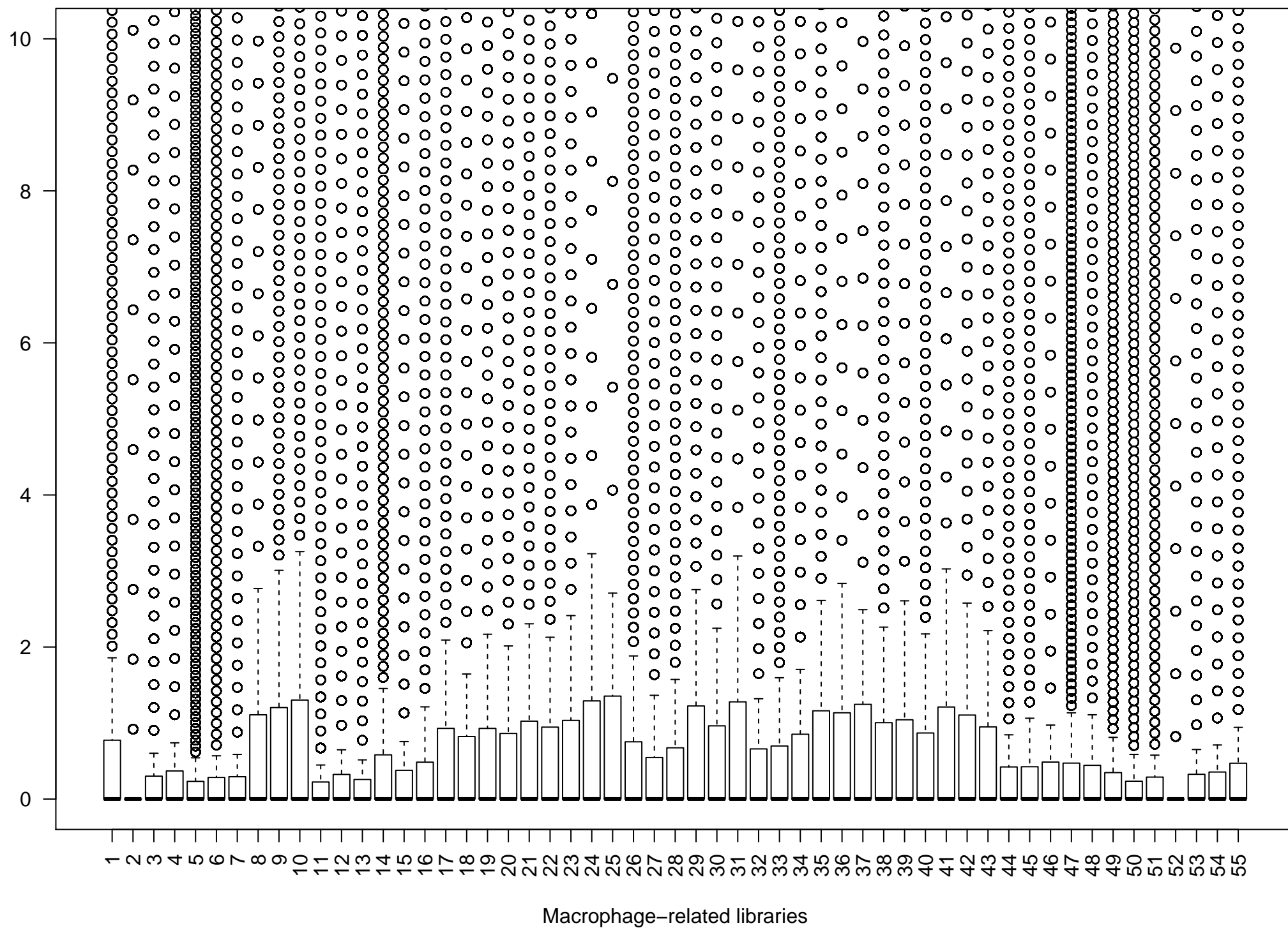

Supplement: Additional file 5: Figure S2. — Distribution of FANTOM5 human promoters’ expression – mappings proximal or distant from pig CAGE CTSS clusters. S2A: FANTOM5 human promoter mapped with proximal (<=2 Kb) pig CAGE CTSS cluster. S2B: FANTOM5 human promoter mapped without a nearby pig CAGE CTSS cluster (>2 Kb). The y-axis shows the FPKM expression values; the x-axis shows the number corresponding to each of the 55 FANTOM5 monocyte and macrophage libraries as described in the Additional file 6: Table S4. (ZIP 282 kb) [file 12864_2015_2111_MOESM5_ESM.zip › Supplementary_Figure_S2B.pdf]

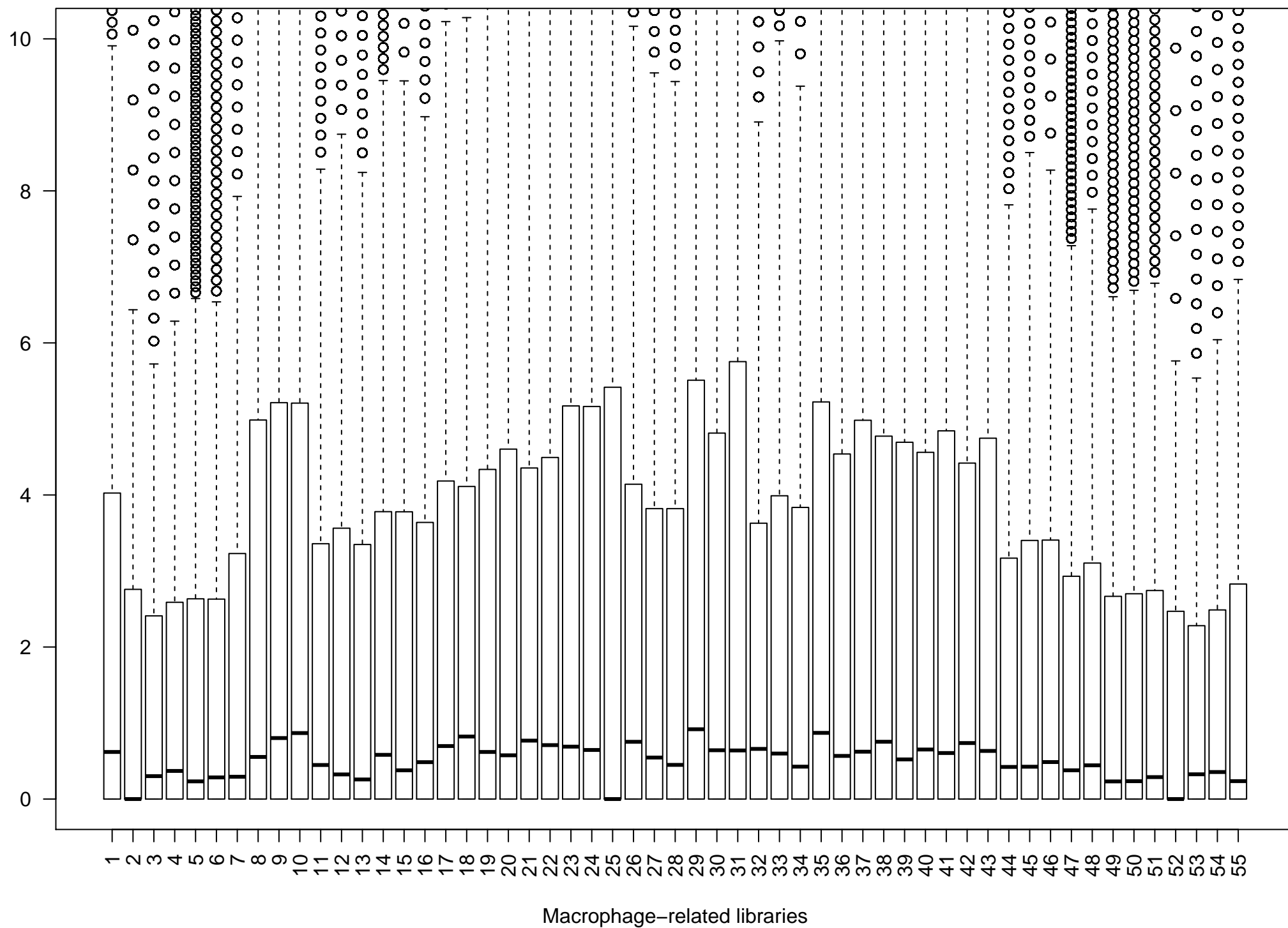

Supplement: Additional file 5: Figure S2. — Distribution of FANTOM5 human promoters’ expression – mappings proximal or distant from pig CAGE CTSS clusters. S2A: FANTOM5 human promoter mapped with proximal (<=2 Kb) pig CAGE CTSS cluster. S2B: FANTOM5 human promoter mapped without a nearby pig CAGE CTSS cluster (>2 Kb). The y-axis shows the FPKM expression values; the x-axis shows the number corresponding to each of the 55 FANTOM5 monocyte and macrophage libraries as described in the Additional file 6: Table S4. (ZIP 282 kb) [file 12864_2015_2111_MOESM5_ESM.zip › Supplementary_Figure_S2A.pdf]

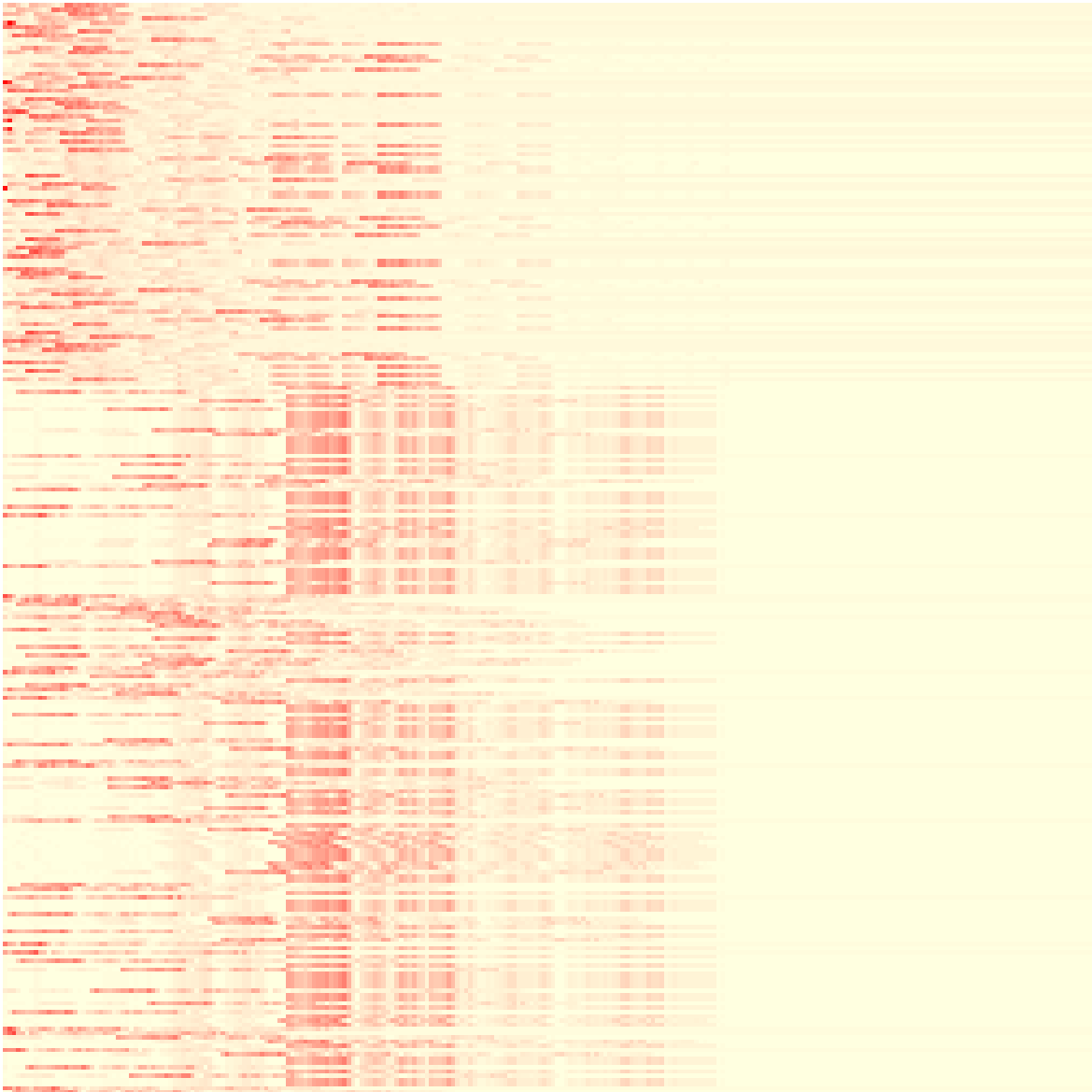

w2 w12 w22 w32 w42 w52 w62 w72 w82 w92 w102 w112 w122 w132 w142 w152 w162 w172 w182 w192 w202 w212 w222 w232 w242

Supplement: Additional file 8: Figures S4A-S4U. — Heatmaps of pig RNA-Seq coverage across FANTOM5 human promoters mapped to the pig genome. Heatmaps showing the RNA-Seq coverage across successive 100 bp genomic windows spanning 5 Kb upstream and 20 Kb downstream of each mapped FANTOM5 human promoter (midpoint of the mapped region taken as reference). FANTOM5 promoter IDs are stacked on the y-axis (labels not displayed). The x-axis corresponds to the successive 100 bp genomic windows, with the window numbered 52 corresponding to the promoter midpoint region. Only those promoters with a minimum of 11 reads across the first 100 windows (covering the midpoint location) were included (i.e. 114,130 promoters). Figures S3A to S3U correspond to heatmaps for the porcine chromosomes: 1–18, X, Y and MT respectively. (ZIP 78361 kb) [file 12864_2015_2111_MOESM8_ESM.zip › Supplementary_Figure_S4U_chr_MT.pdf]

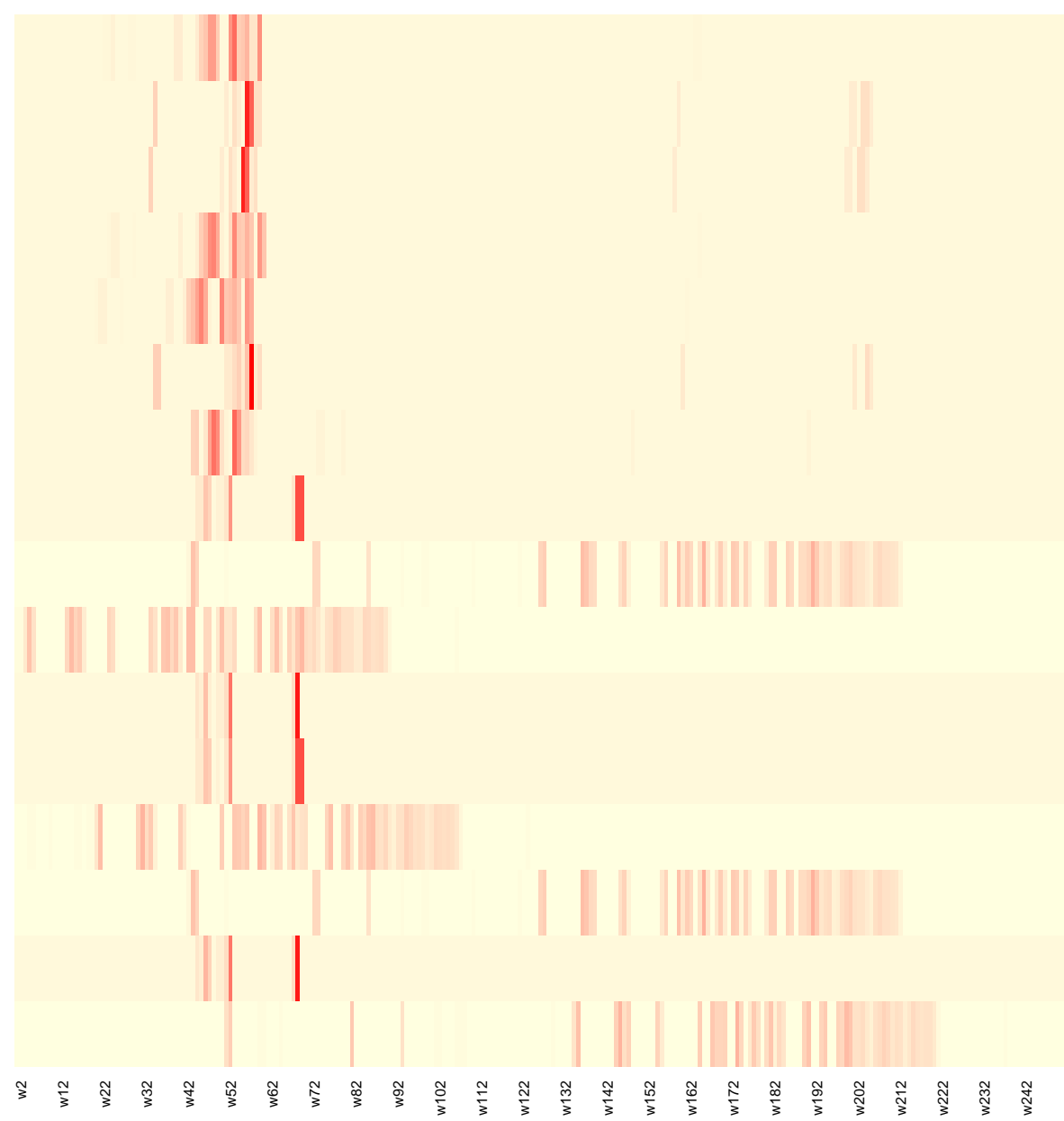

Supplement: Additional file 8: Figures S4A-S4U. — Heatmaps of pig RNA-Seq coverage across FANTOM5 human promoters mapped to the pig genome. Heatmaps showing the RNA-Seq coverage across successive 100 bp genomic windows spanning 5 Kb upstream and 20 Kb downstream of each mapped FANTOM5 human promoter (midpoint of the mapped region taken as reference). FANTOM5 promoter IDs are stacked on the y-axis (labels not displayed). The x-axis corresponds to the successive 100 bp genomic windows, with the window numbered 52 corresponding to the promoter midpoint region. Only those promoters with a minimum of 11 reads across the first 100 windows (covering the midpoint location) were included (i.e. 114,130 promoters). Figures S3A to S3U correspond to heatmaps for the porcine chromosomes: 1–18, X, Y and MT respectively. (ZIP 78361 kb) [file 12864_2015_2111_MOESM8_ESM.zip › Supplementary_Figure_S4T_chr_Y.pdf]

w2  
w12  
w22  
w32  
w42  
w52  
w62  
w72  
w82  
w92  
w102  
w112  
w122  
w132  
w142  
w152  
w162  
w172  
w182  
w192  
w202  
w212  
w222  
w232  
w242

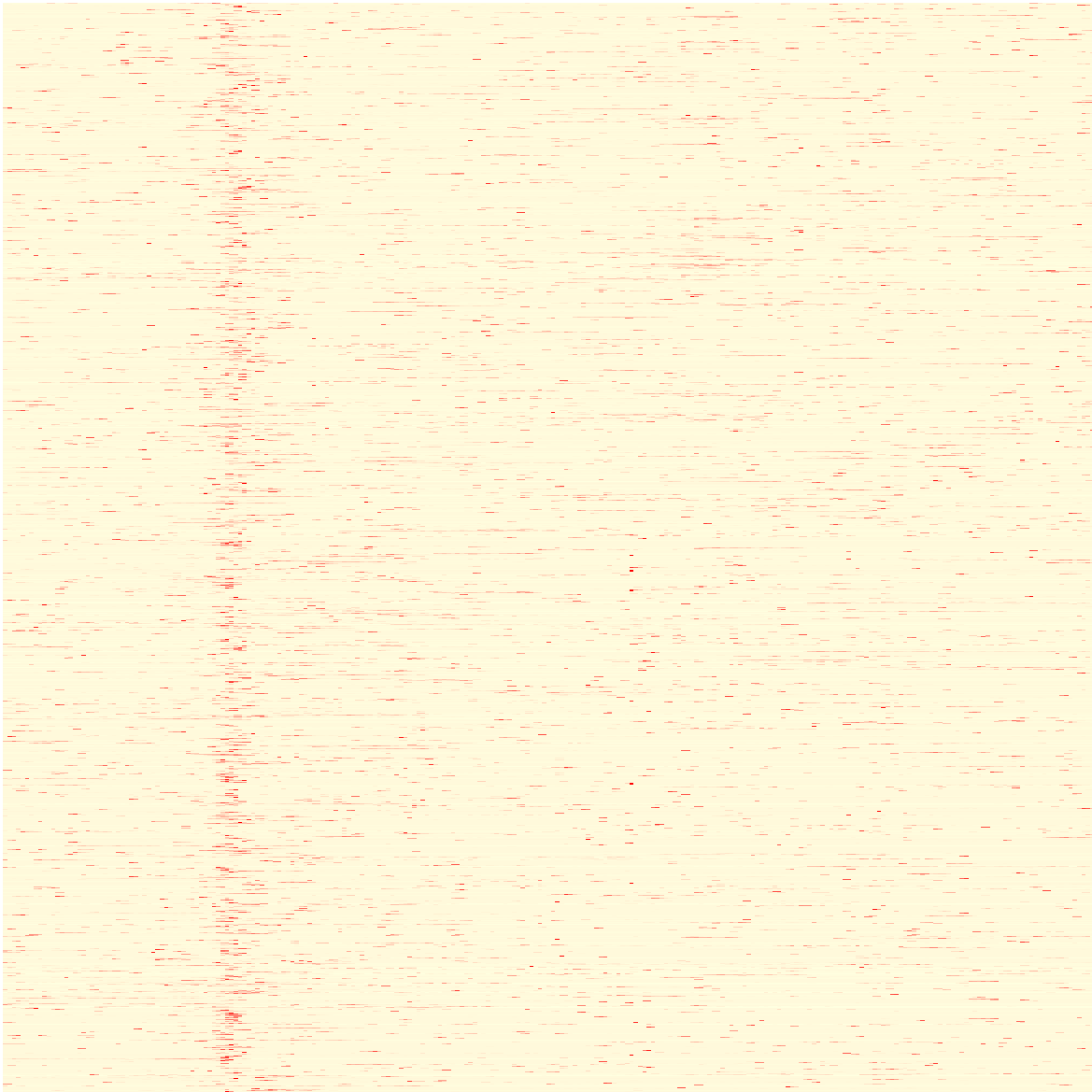

Supplement: Additional file 8: Figures S4A-S4U. — Heatmaps of pig RNA-Seq coverage across FANTOM5 human promoters mapped to the pig genome. Heatmaps showing the RNA-Seq coverage across successive 100 bp genomic windows spanning 5 Kb upstream and 20 Kb downstream of each mapped FANTOM5 human promoter (midpoint of the mapped region taken as reference). FANTOM5 promoter IDs are stacked on the y-axis (labels not displayed). The x-axis corresponds to the successive 100 bp genomic windows, with the window numbered 52 corresponding to the promoter midpoint region. Only those promoters with a minimum of 11 reads across the first 100 windows (covering the midpoint location) were included (i.e. 114,130 promoters). Figures S3A to S3U correspond to heatmaps for the porcine chromosomes: 1–18, X, Y and MT respectively. (ZIP 78361 kb) [file 12864_2015_2111_MOESM8_ESM.zip › Supplementary_Figure_S4R_chr_18.pdf]

w2  
w12  
w22  
w32  
w42  
w52  
w62  
w72  
w82  
w92  
w102  
w112  
w122  
w132  
w142  
w152  
w162  
w172  
w182  
w192  
w202  
w212  
w222  
w232  
w242

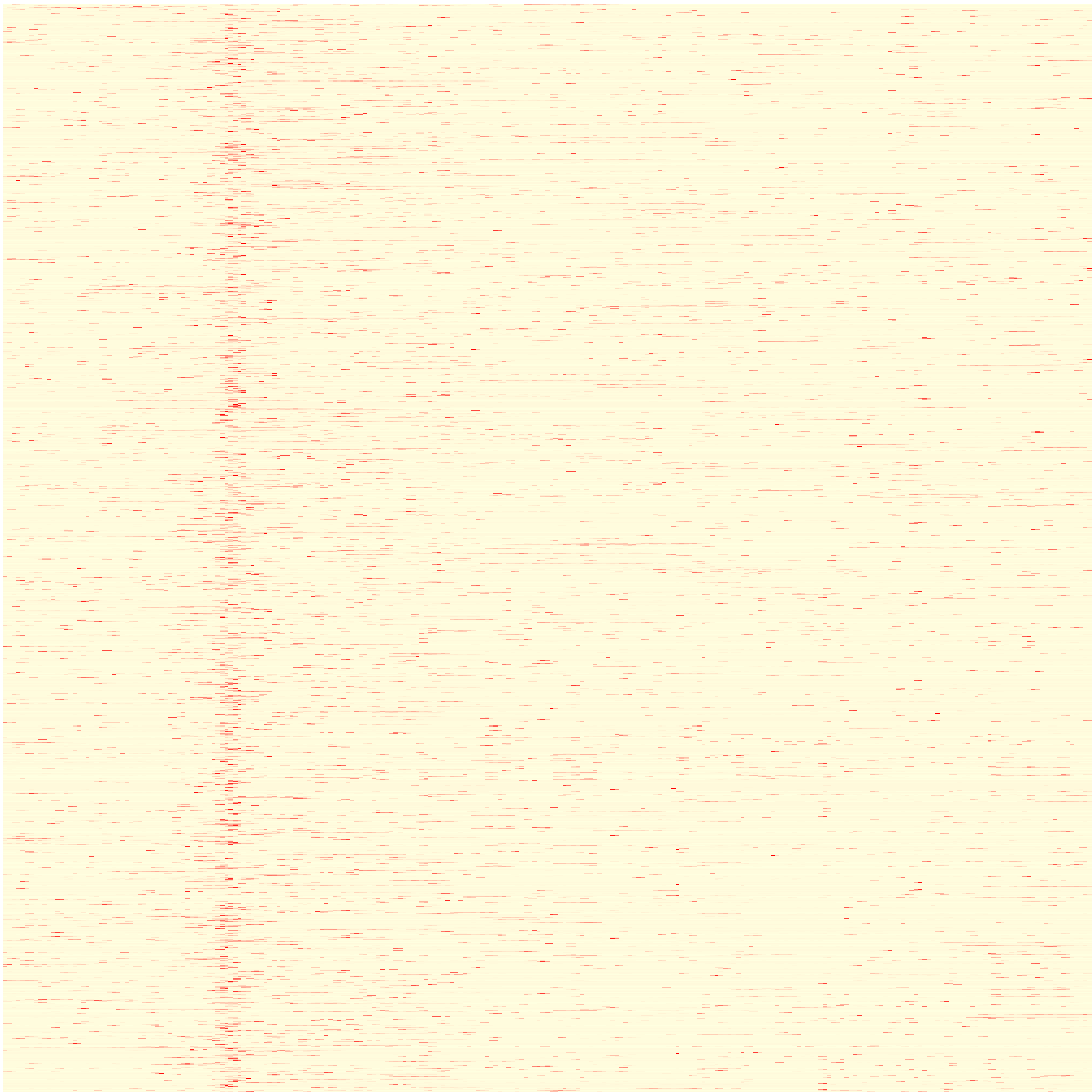

Supplement: Additional file 8: Figures S4A-S4U. — Heatmaps of pig RNA-Seq coverage across FANTOM5 human promoters mapped to the pig genome. Heatmaps showing the RNA-Seq coverage across successive 100 bp genomic windows spanning 5 Kb upstream and 20 Kb downstream of each mapped FANTOM5 human promoter (midpoint of the mapped region taken as reference). FANTOM5 promoter IDs are stacked on the y-axis (labels not displayed). The x-axis corresponds to the successive 100 bp genomic windows, with the window numbered 52 corresponding to the promoter midpoint region. Only those promoters with a minimum of 11 reads across the first 100 windows (covering the midpoint location) were included (i.e. 114,130 promoters). Figures S3A to S3U correspond to heatmaps for the porcine chromosomes: 1–18, X, Y and MT respectively. (ZIP 78361 kb) [file 12864_2015_2111_MOESM8_ESM.zip › Supplementary_Figure_S4K_chr_11.pdf]
