# Supplementary material for: Identification and annotation of conserved promoters and macrophage-expressed genes in the pig genome
Source: BMC Genomics. 2015 Nov 18;16:970. doi: 10.1186/s12864-015-2111-2 (PMC4652390; doi:10.1186/s12864-015-2111-2)

Distribution of the number of tags per pig CAGE CTSS cluster
with/without a nearby mapped FANTOM5 (F5) human promoter

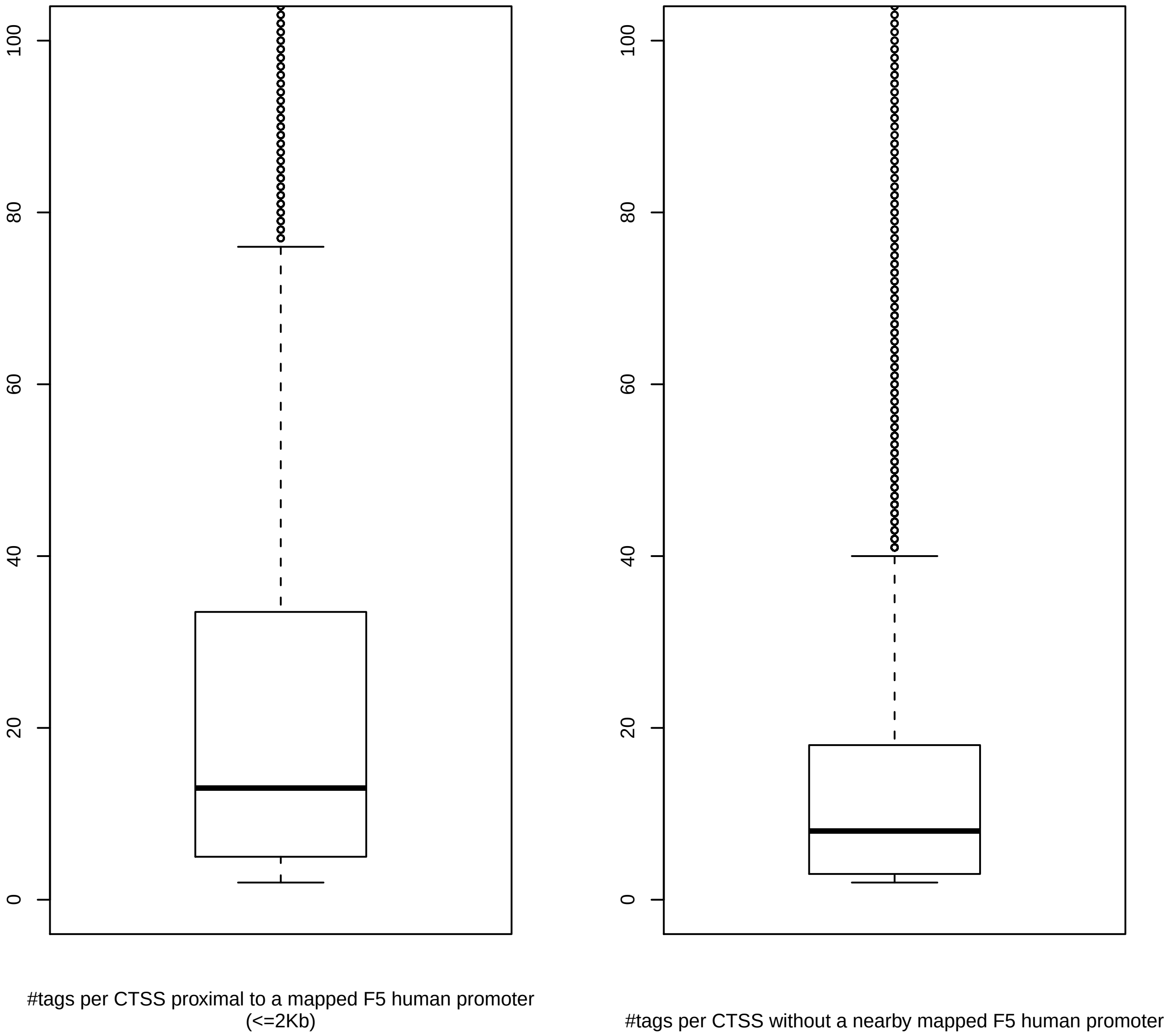

Supplement: Additional file 7: Figure S3. — Distribution of tags per pig CAGE CTSS cluster with/without proximal mapped FANTOM5 human promoter. Boxplots of the number of tags per pig CAGE CTSS cluster with a proximal mapped FANTOM5 human promoter –at most 2 Kb away- (left) and without a nearby mapped FANTOM5 human promoter (right). (PDF 167 kb) [file 12864_2015_2111_MOESM7_ESM.pdf]
