# Supplementary material for: Identification and annotation of conserved promoters and macrophage-expressed genes in the pig genome
Source: BMC Genomics. 2015 Nov 18;16:970. doi: 10.1186/s12864-015-2111-2 (PMC4652390; doi:10.1186/s12864-015-2111-2)
Supplement: Additional file 8: Figures S4A-S4U. — Heatmaps of pig RNA-Seq coverage across FANTOM5 human promoters mapped to the pig genome. Heatmaps showing the RNA-Seq coverage across successive 100 bp genomic windows spanning 5 Kb upstream and 20 Kb downstream of each mapped FANTOM5 human promoter (midpoint of the mapped region taken as reference). FANTOM5 promoter IDs are stacked on the y-axis (labels not displayed). The x-axis corresponds to the successive 100 bp genomic windows, with the window numbered 52 corresponding to the promoter midpoint region. Only those promoters with a minimum of 11 reads across the first 100 windows (covering the midpoint location) were included (i.e. 114,130 promoters). Figures S3A to S3U correspond to heatmaps for the porcine chromosomes: 1–18, X, Y and MT respectively. (ZIP 78361 kb) [file 12864_2015_2111_MOESM8_ESM.zip › Supplementary_Figure_S4S_chr_X.pdf]

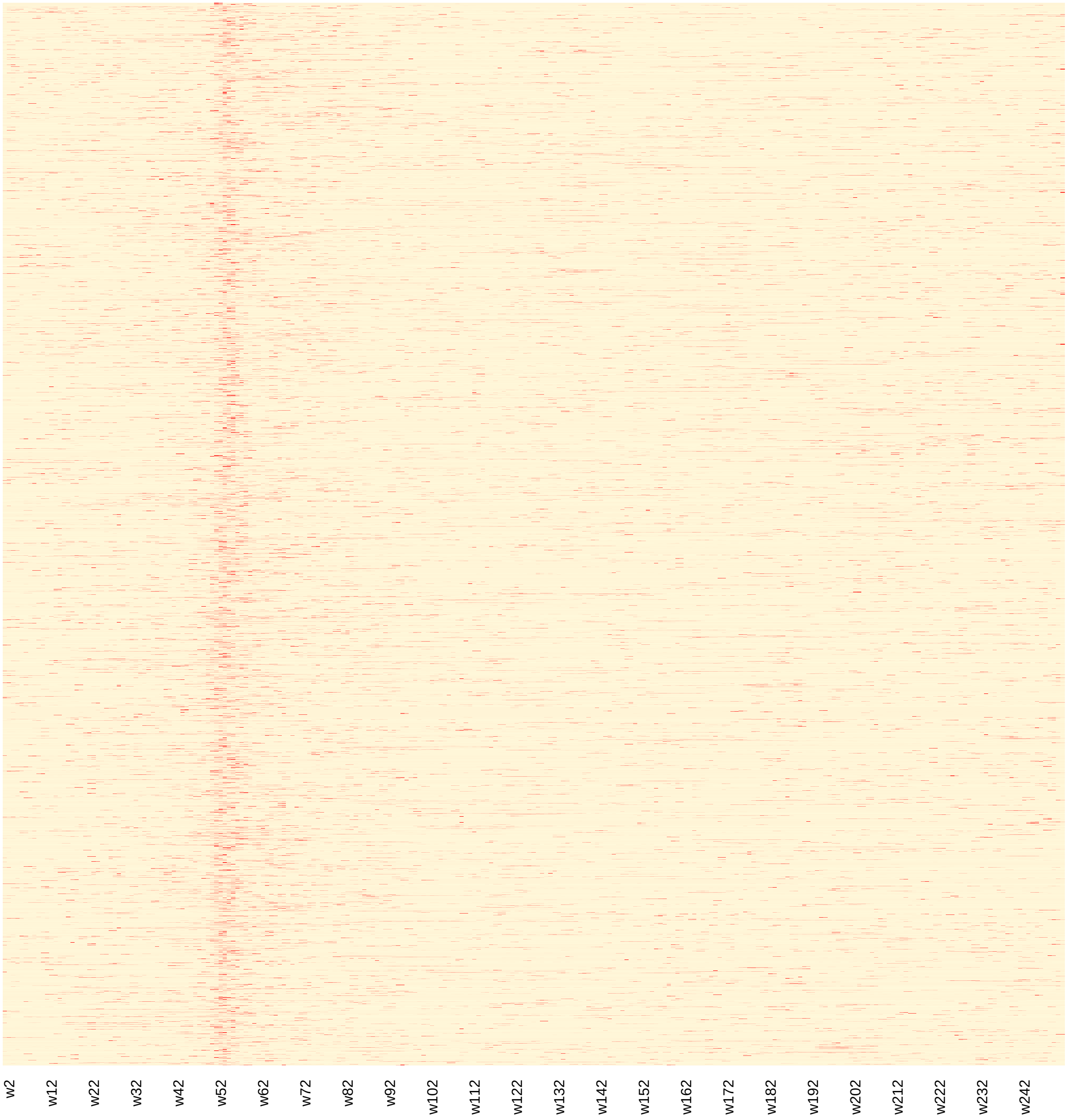
w2
w12
w22
w32
w42
w52
w62
w72
w82
w92
w102
w112
w122
w132
w142
w152
w162
w172
w182
w192
w202
w212
w222
w232
w242
